# Beta diversity differs among hydrothermal vent systems: Implications for conservation

**DOI:** 10.1371/journal.pone.0256637

**Published:** 2021-08-26

**Authors:** Thomas N. Giguère, Verena Tunnicliffe

**Affiliations:** 1 School of Earth & Ocean Sciences, University of Victoria, Victoria, British Columbia, Canada; 2 Department of Biology, University of Victoria, Victoria, British Columbia, Canada; National Cheng Kung University, TAIWAN

## Abstract

Deep-sea hydrothermal vent habitats are small, rare and support unique species through chemosynthesis. As this vulnerable ecosystem is increasingly threatened by human activities, management approaches should address biodiversity conservation. Diversity distribution data provide a useful basis for management approaches as patterns of β-diversity (the change in diversity from site to site) can guide conservation decisions. Our question is whether such patterns are similar enough across vent systems to support a conservation strategy that can be deployed regardless of location. We compile macrofaunal species occurrence data for vent systems in three geological settings in the North Pacific: volcanic arc, back-arc and mid-ocean ridge. Recent discoveries in the Mariana region provide the opportunity to characterize diversity at many vent sites. We examine the extent to which diversity distribution patterns differ among the systems by comparing pairwise β-diversity, nestedness and their additive components. A null model approach that tests whether species compositions of each site pair are more or less similar than random provides insight into community assembly processes. We resolve several taxonomic uncertainties and find that the Mariana arc and back-arc share only 8% of species despite their proximity. Species overlap, species replacement and richness differences create different diversity distributions within the three vent systems; the arc system exhibits much greater β-diversity than both the back-arc and mid-ocean ridge systems which, instead, show greater nestedness. The influence of nestedness on β-diversity also increased from the arc to back-arc to ridge. Community assembly processes appear more deterministic in the arc and ridge systems while back-arc site pairs deviate little from the null expectation. These analyses reflect the need for a variety of management strategies that consider the character of diversity distribution to protect hydrothermal vents, especially in the context of mining hydrothermal deposits.

## Introduction

Conservation of biodiversity underlies policies for sustainable approaches to live with and use natural systems [1]. Basic information on species arrangement over the landscape is fundamental to detecting change and predicting responses to threats [2]. Asaad *et al*. [3] describe eight relevant ecological criteria requiring species occurrence data, including endemicity, geographic range and species richness. Local species occurrences can differ among sites, both in richness (alpha(α)-diversity) and composition. This variability is captured as beta(β)-diversity, the facet of regional diversity encompassing differences among local assemblages [4]. Socolar *et al*. [5] and Carlos-Júnior *et al*. [6] outline use of β-diversity in conservation, including management planning, such as choosing which sites to protect.

As many ecological phenomena can shape β-diversity, patterns can reveal key processes. By separating β-diversity into its components, ecologists can both describe diversity patterns and test hypotheses regarding mechanisms that shape them [7–9]. The specific formulation to identify components is debated (e.g., [10–12]), but we find the recent SET framework [13] derives a clear scenario. This framework introduces the concept of *pairwise pattern components* (PPCs) that isolate the community response pattern between every pair of sites examined for species presence/absence. It also incorporates the role of nestedness in the concepts of ‘intersection of nestedness and β-diversity’ and ‘the relative complement of nestedness in β-diversity’. Partitioning β-diversity into its components helps to describe the regional pattern and to support hypotheses around the mechanisms that shape diversity distributions [7]. A conservation approach can target the underlying mechanism if one component dominates a region. For example, where nestedness is low, selection pressures have likely driven species substitution (e.g., [9]), and conservation may need to target several representative sites rather than one larger area. Combined with the null model approach of Raup and Crick [14], these tools provide insight into the processes shaping differences between sites [15].

The remoteness of the deep ocean no longer buffers human impacts. Less than 10% of the deep ocean is classified as “wilderness”, with only 0.5% in Marine Protected Areas [16]. As climate change, plastic pollution and ocean dumping impacts increase, exploitation for food, natural products and mineral resources is expanding in the deep sea [17–19]. So far, hydrothermal vents remain relatively untouched, as reflected in the similar contributions of common and rare species to functional diversity, in contrast to disrupted terrestrial systems [20]. However, the seafloor massive sulphides formed at vents has attracted mining interests despite the minimal projected economic returns [21]. Beyond national jurisdictions, the International Seabed Authority has awarded seven exploration contracts for massive sulphides to date (www.isa.org.jm/exploration-contracts/polymetallic-sulphides). Immediate development of management plans should include, *inter alia*, designating conservation areas (e.g., [22]) for which knowledge of species diversity and distributions is a critical component. As most vent studies focus on single sites, regional β-diversity is poorly understood, although a few applications have revealed local [23, 24] to broader patterns [25].

The reduced compounds dissolved in vent water sustain microbial chemosynthesis and associated lush animal communities; most animals at hot vents are known nowhere else [26]. While vents occur on spreading centres and subsea volcanoes in every ocean, and appear to be abundant [27], the habitat extent is highly constrained to fluid outlets; consequently, the global vent ecosystem is very small [28]. Vents are insular habitats distributed along geologic structures where inter-site distances can exceed 100s of km. Site stability varies from frequent volcanic disruption [29] to millennia-long fluid delivery [30]. Nonetheless, vent habitats tend to be characterized as unstable, disturbed, and short-lived with inhabitants adapted to such conditions [31–33], where a single management strategy may seem appropriate. Despite common basic characteristics, similar ecosystem types vary among regions in their β-diversity patterns [34, 35], thus conservation approaches for vent habitats may need to target differing underlying causes.

**Fig 1:**
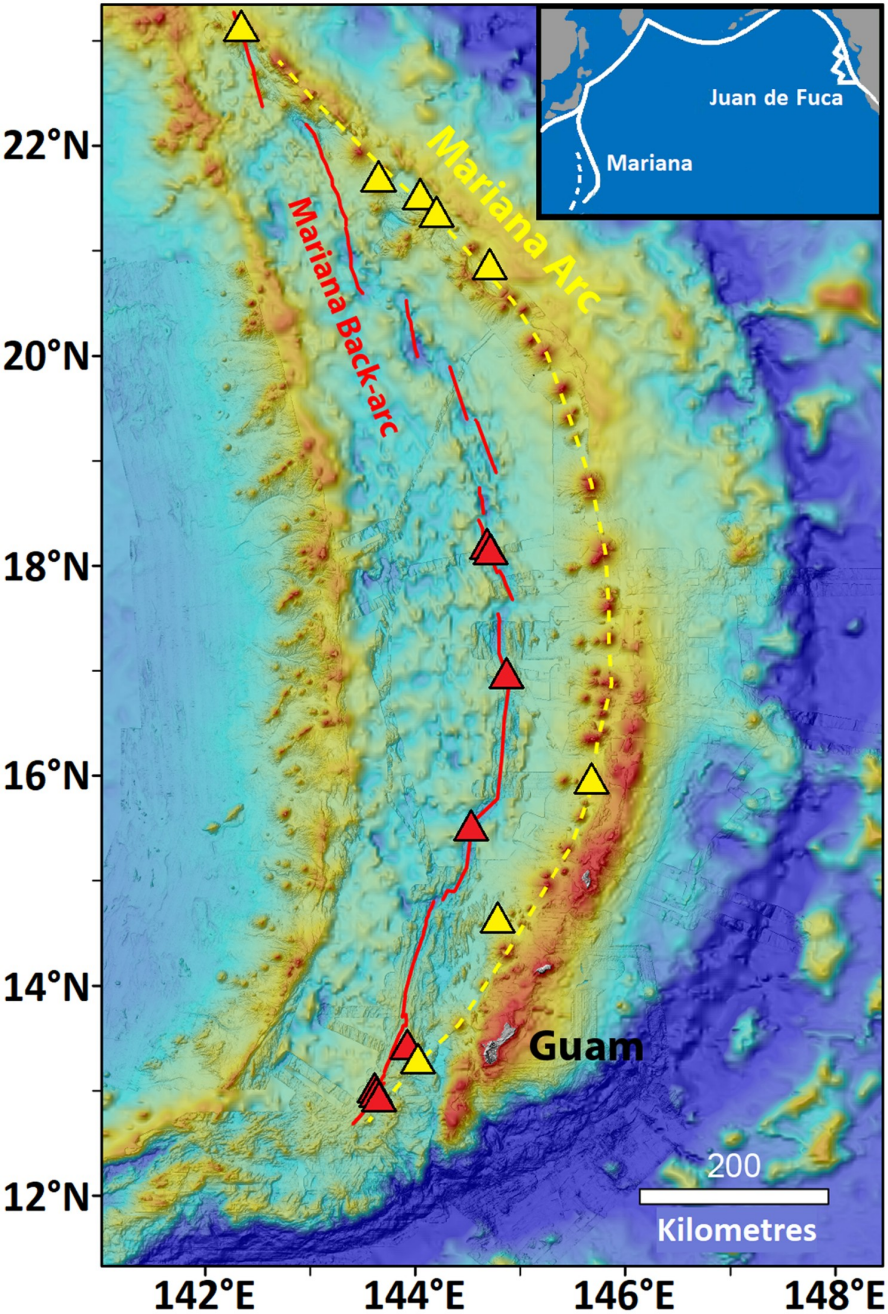
Map of the Mariana region and its known hydrothermal vent sites. The Mariana tectonic region includes back-arc and volcanic arc vent systems. The red and yellow lines trace the back-arc and arc respectively, and coloured triangles indicate locations of hydrothermal vent sites in this study. From north to south, the red triangles represent Alice Springs/Illium, Burke (overlapping), Hafa Adai, Perseverance, Forecast, then Snail, Archaean and Urashima/Pika (overlapping). From north to south, the yellow triangles represent Nikko, Kasuga-2, NW Eifuku, Daikoku, Chamorro, E Diamante, NW Rota and Seamount X. The last site lies only 20 km from Forecast. Inset shows locations of the Mariana and Juan de Fuca vent systems in the North Pacific. Map produced by WW Chadwick using data from GEBCO (British Oceanographic Data Centre) and from NOAA surveys.

Vent species are distributed among biogeographic regions following vicariant patterns related to tectonic history [26, 36]. The region lying westward of the Mariana Trench includes the Mariana back-arc spreading centre and the Mariana volcanic arc (Fig 1). The 1500 km long Mariana arc has a northward extension as the Izu-Bonin arc. Many of the submarine volcanoes are hydrothermally active with fluids enriched in CO_2_ and SO_2_ [37–39] and notable variability in both fluid and faunal characteristics among sites [40]. Faunal diversity along the arc is incompletely documented [41]. The 1300 km long Mariana back-arc is located in the Mariana Trough where the spreading axis is highly segmented; magmatic influence from the adjacent arc increases from central to southern segments [42] where hydrothermal fluids reflect input from magmatic volatiles [43]. Kojima and Watanabe [44] review faunal samples from 1987 to 2010 at six sites on these segments and describe a distinct back-arc fauna with low similarity to the adjacent Mariana-Izu-Bonin arc system. Many faunal records from the Mariana back-arc are not resolved to species level, and there is low confidence in some names assigned from decades past. Some Mariana species names appear in lists from other biogeographic regions suggesting broad distributions, but closer study may detect greater regionalism (e.g., [45]).

Since 2004, the Vents Program and Ocean Exploration Program of the National Oceanic and Atmospheric Administration (NOAA) have supported research cruises in the region. One outcome of the discoveries was the declaration, in 2009, of the Mariana Trench Marine National Monument (MTMNM) that includes the Volcanic Unit encompassing the known vent sites. Two recent missions investigated hydrothermalism in the southern Mariana back-arc. The first located water column signatures from 19 possible seafloor vent sites [46]. The densest cluster occurs in the south where distance to the arc falls and spreading rate increases to 56 mm yr^-1^. Work with a remotely operated vehicle (ROV) the following year confirmed two new vent sites in the south-central axis plus a newly eruptive site where venting was short-lived [47].

Our study documents diversity at the new back-arc sites and compiles a regional species check-list for all known sites in the Mariana region. We examine the hypothesis that there is an along-strike faunal shift within the Mariana back-arc using measures of α- and β-diversity and explore some potential drivers. Lastly, we investigate the relative partitions of β-diversity in three hydrothermal systems of similar extent but differing geological settings: back-arc, volcanic arc and mid-ocean ridge. This last region lies in the Northeast Pacific: the Juan de Fuca-Explorer Ridge complex. We examine the extent to which these hydrothermal systems show similar patterns given that vents host relatively low diversity communities with a similar basis in chemosynthesis. We assess the extent to which such analyses can inform conservation management of this unusual, but vulnerable, ecosystem.

## Methods

Collection in the Mariana Trench Marine National Monument was conducted with on board oversight by the National Oceanic and Atmospheric Administration of the USA. Collection on the Juan de Fuca Ridge was conducted under permit #16-OPAC-00002EHV.

### Study locations

A vent ‘system’ is an array of vent sites on a distinct geologic structure, such as a spreading centre or volcanic arc. A ‘site’ refers to a broad area of venting influenced by an underlying geological process. A site may have more than one vent ‘field’, each of which appears to have a discrete heat source. Vents form around discrete fluid outlets on the seafloor. In our study, fields less than 3 km apart were combined into one site.

#### Mariana back-arc spreading centre

In 2016, the Schmidt Ocean Institute Research Vessel *Falkor* mapped and sampled four vent sites from 15.5°N to 18.2°N with the Remotely Operated Vehicle (ROV) *SuBastian*: two newly discovered sites, Perseverance and Hafa Adai (Fig 1), and two previously visited sites, Alice Springs/Illium and Burke [48]. We sampled three additional sites further south with ROV *Jason-2* (Forecast in 2006, Snail and Urashima/Pika in 2014); other species reports from these sites, plus Archaean, derive from Kojima and Watanabe [44]. Table 1 presents site characteristics.

**Table 1:** Environmental variables for the Mariana back-arc vent sites.

	Alice Springs/Illium	Burke	Hafa Adai	Perseverance	Forecast	Snail	Archaean	Urashima/Pika
**Coordinates**	18°12.71’N 144°42.45’E	18°10.95’N 144°43.19’E	16°57.68’N 144°52.15’E	15°28.80’N 144°30.46’E	13°24’N 143°55’E	12°57.20’N 143°37.20’E	12°56’N 143°38’E	12°55.10’N 143°38.90’E
**Depth (m)**	3597	3630	3279	3910	1470	2850	2990	2956
**Distance from arc (km)**	109	108	101	97	23	11	8	6
**Distance to next site south (km)**	3.5	136.7	169.2	239.9	59.1	2.7	2.3	NA
**Max temperature (°C)**	165 (287^a^)	50	345	264	136 (210^b^)	214 (248^b^)	(345^b^)	196 (330^b^)
**Est venting area (m** ^**2**^ **)**	2270	2625	5165	510	2510^c^	1183^c^	1840^c^	1995^c^

Vent sites of the Mariana back-arc spreading centre and their respective geographic coordinates. The environmental variables included were those suspected to drive the diversity distribution patterns in this vent system; therefore, these variables were analyzed with the associated α- and β-diversity data of each site.

The highest temperature measured during the “Ring of Fire” cruises is shown with past highest temperatures in brackets.

^a^ Maximum temperature measurement from [49].

^b^ Maximum temperature measurements from [43].

^c^ Estimated venting area using JAMSTEC imagery.

#### Mariana volcanic arc

Biological sampling at eight seamounts with vents was uneven as the six expeditions (2004–2014) differed in objectives. Descriptions of all sites are available [37, 40, 43, 50–52], including in cruise reports (www.pmel.noaa.gov). Vent sites (Fig 1) are located on seamount summits, some of which are recently volcanically active (NW Rota, Daikoku). A key feature of the arc vents is variability in venting characteristics in which values for pH, CO_2_, SO_2_, H_2_S and S^0^ differed markedly among sites [39, 52].

#### Juan de Fuca ridge

We use data from seven sites along the Juan de Fuca and Explorer Ridges in the Northeast Pacific for comparison (Fig 1 inset). This mid-ocean ridge system has several large vent sites including a central active volcano (Axial Seamount), extensive fields of black smokers (Endeavour) and a sedimented massive sulphide deposit (Middle Valley). Data derive from many studies (e.g. [25, 53–56]) in addition to work at individual vents and in systematic descriptions. Vent assemblages of the southern and northern sites (South Cleft and Explorer) are less well assessed. While Explorer Ridge is separated from the Juan de Fuca by a 150 km long transform fault, we treat these seven sites as a single system.

### Data collection

In general, methods in all locations and years were similar. Pilots of the ROVs *ROPOS*, *Jason-2* or *SuBastion* executed the collections as guided by scientists. Imagery acquisition used digital still cameras and video cameras of increasing quality over the years to the current high definition systems. Due to rough terrain on different habitat types, a quantitative approach was not possible. Manipulator arms collected animals and substratum either directly or with scoops to deposit them in closable, sealed boxes. A suction sampler pulled mobile or small animals into swivelling sealed jars. Sampling was more consistent among sites at the JdF and, largely, at the Mariana back-arc as most diversity was associated with one foundation species. However, for the Mariana arc sites, the high variability in sample retrieval methods did not support sample standardization for richness estimates. At some sites, collection was not possible at all major vents. Thus, diversity is very likely higher than represented, especially for Nikko and Daikoku. Nonetheless, as similar collection approaches were used, an effort to establish overall patterns in diversity distributions is warranted.

Samples were stored in 75–80% ethanol or 7% buffered formalin on board ship. On shore, a 1 mm sieve separated macrofauna from meiofauna for complete sorting of all samples. Identifications followed published descriptions and consultations with systematics experts, some of whom used molecular approaches. We sent some specimens from the Mariana arc and back-arc to BOLD, the Centre for Biodiversity Genomics (boldsystems.org), to compare COI barcodes with previous work.

Dive imagery was reviewed for the Mariana back-arc, including that available from the JAMSTEC-EDI system (www.godac.jamstec.go.jp/jedi/e/) for missions conducted with Japanese vehicles. Larger species and several smaller species in high definition are distinctive and added to the records. We include taxa from Kojima and Watanabe [44] who summarize all reports to that time. Of the 47 macrofauna listed in their study, 18 have species identities. We cannot match the remainder to our species list as different authors may have made conflicting designations, especially those listed as *con forma* (cf.) or “sp.”. Thus, we use a conservative list in which species are identified consistently along the back-arc for our analyses. For the Mariana arc, our data are supplemented for the northern seamounts from Watanabe *et al*. [41].

### Data analysis

#### Diversity

Following Whittaker [4], α- and γ-diversity represent “local” and “regional” species richness, respectively, where the number of species at a site determines the α-diversity value, and γ-diversity is the number of species present at all sites in the system. We also make a larger ‘region’ by combining all Mariana data in a separate analysis. We used the Chao1 estimator [57] in PAST 2.17 [58] to estimate an upper species number for the four Mariana back-arc vent sites sampled in 2016. Only macrofaunal vent-associated species are used.

As data are restricted to site-by-species presence matrices, we use the Jaccard family of indices [59, 60]. Using the POD and SET frameworks [12, 13, 61], we identify the diversity patterns in each vent system by quantifying the *pairwise pattern components* (PPCs) using the ‘beta.div.comp’ function in the ‘adespatial’ R package [62, 63]. The PPCs are represented as species *overlap* (O_J_), *richness difference* (D_J_) and *replacement* (R_J_), which can occur singly or in combination [13]. As they correspond with the SDR-simplex indices developed by Podani and Schmera [61], we generated simplex plots for each vent system using the ‘TernaryPlot’ function in the ‘Ternary’ R package [64]. We calculate β_J_-diversity (β_J_ = 1 –O_J_ = R_J_ + D_J_) and nestedness (N_J_ = 1 –R_J_ = O_J_ + D_J_). Dendrograms for each vent system, generated using the ‘average’ method and ‘hclust’ function in the ‘vegan’ R package [63, 65], illustrate relative similarities of vent sites. We calculated the LCBD (Local Contribution to Beta Diversity) using the ‘beta.div’ function in the ‘adespatial’ R package [62, 63, 66] to determine which sites contribute most to the overall β_J_-diversity of each system.

Following the SET framework, we calculate the intersection (I_J_) of nestedness and β-diversity and the relative complement (RC_J_) of nestedness in β-diversity. Given that D_J_ is used to calculate both β_J_ and N_J_, the relativized richness difference is the intersection of nestedness and β-diversity (D_J_ = I_J_), but only when O_J_ > 0; otherwise, I_J_ = 0 and RC_J_ = 1 [13]. The β_ratio_ (I_J_ / β_J_) for each pair of sites is an indication of which additive component plays the dominant role in shaping β-diversity [67].

The pairwise Raup-Crick dissimilarity index (β_RC_) is a measure of the probability that an observed β_J_-diversity value would occur by chance using a null model that controls for richness differences [15, 68]. β_RC_ values falling beyond the 95% confidence intervals (CIs) exhibit significant deviation from the null expectation and may implicate deterministic mechanisms in community assembly [15]. We calculated β_RC_ values using the ‘raupcrick’ function in the ‘vegan’ R package [63] and converted output values to a scale of negative one to positive one [15]. To illustrate the similarities of vent sites relative to the expectation of random assembly (β_RC_), we generated non-metric multi-dimensional scaling (nMDS) plots generated using the ‘metaMDS’ function in the ‘vegan’ R package [63, 65].

#### Between-system comparisons

Since the Mariana volcanic arc and back-arc share some species, we combine data to calculate PPC and β_RC_ values and thereby illustrate dissimilarity patterns over the entire Mariana region. We also compared the pairwise β_J_-diversity, its additive components, the pairwise nestedness and β_RC_-diversity values among the three vent systems [69], while acknowledging inherent limitations [70]. The method allows application of significance tests through permutation ANOVA and permutation t-tests using the ‘perm.oneway.anova’ function in the ‘rcompanion’ R package [71] and the ‘perm.t.test’ function in the ‘RVAideMemoire’ R package [72], respectively; the probability threshold for all significance tests was set at 0.05.

#### Environmental analyses

We examined environmental variables that may affect diversity on the Mariana back-arc (Table 1); similar consistent data were not available for the volcanic arc or Juan de Fuca systems. Depth is a well-known diversity driver [73]. Distance from the volcanic arc affects magmatic activity and topography [42, 74], therefore influencing hydrothermalism and local currents for dispersing larvae. Distance between sites affects connectivity, while the maximum fluid temperature (measured at any visit) is an indication of relative hydrothermal vigour at the site. Habitat area, another diversity predictor [75], was estimated using maps in cruise reports and, for Forecast, in Fujikura *et al*. [76]. By integrating video footage and dive track maps, we used ImageJ to create polygons over areas with hydrothermal indicators such as venting fluids, bacterial mats, and vent animals.

We calculated Kendall rank correlation coefficients using the ‘cor.test’ function in R [63] to assess associations between α-diversity and each environmental variable over the sites. For the significant explanatory variables (two total), simple regressions and generalized linear models, generated in R using the ‘lm’ function and the ‘glm’ function of the ‘mgcv’ R package [77], determined if the significant correlations were linear. We used dbMEM analysis using the ‘adespatial’ R package [66] to compare the β-diversity values to the environmental variables. As β-diversity values correspond with pairs of sites, relevant environmental variables were pairwise comparisons (differences in depth, arc distance, temperature and area; distance between sites).

## Results

### Mariana back-arc

A brief description and images of the back-arc habitats appears in S1 Text in S1 File. The four sampled sites returned 28 macrofaunal species (Table 2). The hairy snail, *Alviniconcha hessleri*, acts as a foundation species expanding surface area for other species, especially crabs (*Austinograea williamsi*) and shrimp (*Rimicaris vandoverae*). Sampling the snails resulted in the recovery of 16 macrofaunal and four meiofaunal species. Overall, among the 2,038 specimens collected, over 47% were individuals of four species: *A*. *hessleri*, *Neoverruca brachylepadoformis*, *Lepetodrilus* aff. *schrolli* MT and *R*. *vandoverae*. Meiofauna were rare: of the 181 meiofaunal specimens, 71% (128/181) were nematodes, and nearly all the remainder were copepods (mostly dirivultids and harpacticoids).

**Table 2:** Macrofauna collected from the four northern-most vent sites in the Mariana back-arc.

Class	Group	Family	Species	Notes
Anthozoa	Actinaria	Kadosactinidae	*Marianactis bythios* Fautin & Hessler 1989	
Anthozoa	Zoantharia	Epizoanthidae	*Epizoanthus* aff. sp. nov.^a^	1^st^ coll.
Aplacophora	Solenogastres	Simrothiellidae	*Helicoradomenia* sp. nov.	[78]
Bivalvia	Mytilida	Mytilidae	*Bathymodiolus septemdierum*^b^ Hashimoto & Okutani 1994	[79]
Gastropoda	Abyssochrysoidea	Provannidae	*Alviniconcha hessleri* Okutani & Ohta 1988	COI
Gastropoda	Abyssochrysoidea	Provannidae	*Provanna nassariaeformis* Okutani 1990	
Gastropoda	Abyssochrysoidea	Provannidae	*Desbruyeresia marianaensis* (Okutani 1990)	COI
Gastropoda	Abyssochrysoidea	Provannidae	*Desbruyeresia chamorrensis* Chen, Ogura & Okutani 2016	COI
Gastropoda	Lepetellida	Lepetodrilidae	*Lepetodrilus* aff. *schrolli* MT^b^	[80] COI
Gastropoda	Lepetellida	Lepetodrilidae	*Pseudorimula marianae* McLean 1989	COI
Gastropoda	Lottoidea	Pectinodontidae	*Bathyacmaea* sp.^b^	COI
Gastropoda	Cycloneritida	Phenacolepadidae	*Shinkailepas* sp. nov.^a^	
Gastropoda	Neomphaloidea	Neomphalidae	*Symmetromphalus regularis* McLean 1990	
Gastropoda	Neogastropoda	Raphitomidae	*Phymorhynchus wareni*^a^^b^ Sysoev & Kantor 1995	
Hexanauplia	Cirripedia	Neoverrucidae	*Neoverruca brachylepadoformis* Newman 1989	COI
Hexanauplia	Cirripedia	Eolepadidae	*Vulcanolepas verenae*^a^ Watanabe, Chen & Chan 2021	1^st^ coll.
Malacostraca	Decapoda	Alvinocarididae	*Rimicaris vandoverae* (Martin & Hessler 1990)	COI
Malacostraca	Decapoda	Alvinocarididae	*Rimicaris* cf. *variabilis* (Komai & Tsuchida 2015)	COI
Malacostraca	Decapoda	Alvinocarididae	*Rimicaris falkorae* Komai & Giguère 2019	COI
Malacostraca	Decapoda	Bythograeidae	*Austinograea williamsi* Hessler & Martin 1989	COI
Pycnogonida	Pantapoda	Ammotheidae	*Sericosura cochleifovea* Child 1989	
Polychaeta	Errantia	Polynoidae	*Levensteiniella raisae* Pettibone 1989	
Polychaeta	Errantia	Polynoidae	*Lepidonotopodium minutum* Pettibone 1989	
Polychaeta	Errantia	Polynoidae	*Branchinotogluma marianus* (Pettibone 1989)	
Polychaeta	Errantia	Hesionidae	*Sirsoe hessleri* (Blake 1991)	
Polychaeta	Sedentaria	Spionidae	*Laonice* sp. nov.	
Polychaeta	Sedentaria	Alvinellidae	*Paralvinella hessleri* Desbruyères & Laubier 1989	
Polychaeta	Sedentaria	Ampharetidae	*Amphisamytha* sp. nov.^a^^b^	

Abbreviations: 1st coll., first time collected but seen (undescribed) by Hessler and Lonsdale [81]; COI, cytochrome c oxidase subunit I barcode sequence available.

^a^ A gene sequenced by collaborators.

^b^ Identity update from prior report(s).

Our work at the four northern sites resolved several macrofaunal species identities to the regional list. Table 2 notes changes in species names from taxa previously reported in this region including two undescribed species, a *Lepetodrilus* limpet and an *Amphisamytha* polychaete, originally identified with names from other vent provinces [82]. Komai and Giguère [83] distinguish two additional shrimp species from the *R*. *vandoverae* originally noted. A spionid polychaete and mite are new reports, and one galatheid did not match described species. Noting four additional species from prior reports, we find 32 species in the four northern sites; individual-based rarefaction (not shown) on the macrofauna indicates a range for estimated species between 32 and 40 for these sites. Given that the two new sites contained no species that were not also present in Burke or Alice Springs/Illium, the current α-diversity numbers may not increase much.

Table 3 presents diversity numbers for all eight sites (complete list in S4 Table in S1 File) averaging ~20 species per site with Alice Springs/Illium having the greatest number. Six species occurred at all sites, while eight (21%) were found at only one site each. γ-diversity of the system is 39 species.

**Table 3:** Species richness measures for the three study vent systems.

**Mariana Back-Arc (γ = 39)**			**Mariana Volcanic Arc (γ = 45)**			**Juan de Fuca Ridge (γ = 71)**		
599 km			1107 km			565 km		
**Site**	**Abbr**	**α**	**Site**	**Abbr**	**α**	**Site**	**Abbr**	**α**
Alice/Illium^a^	AI	29	Nikko^a^	Nk	12	Explorer^a^	Ex	29
Burke^a^	Bk	23	Kasuga-2	K2	12	Middle Valley^a^	MV	46
Hafa Adai^b^	HA	25	NW Eifuku^a^	NWE	21	Endeavour^a^	En	45
Perseverance^b^	Pv	13	Daikoku^a^	Dk	14	CoAxial	CA	27
Forecast^a^	Fc	20	Chamorro	Ch	5	Axial^a^	Ax	40
Snail^a^	Sn	21	East Diamante^a^	ED	16	North Cleft^a^	NC	28
Archaean^a^	Ar	13	NW Rota	NWR	9	South Cleft	SC	16
Urashima/Pika^a^	UP	15	Seamount X^a^	SX	14			
$\bar{\#}$ *spp/site (sd)*	19.9 (5.8)			12.9 (4.7)			35.0 (11.0)	
γ / $\bar{\alpha }$	1.96			3.49			2.15	

α-diversity for sites on each hydrothermal system. The second line is the along-structure distance between the farthest sites.

Abbreviations: Abbr, abbreviations for vent site names; γ, gamma diversity; α, alpha diversity; γ/$\bar{\alpha }$, “true beta-diversity” *sensu* Whittaker [4].

^a^ The minimum combination of sites that hold all species known in the respective hydrothermal system.

^b^ Newly assessed vent sites.

**Fig 2:**
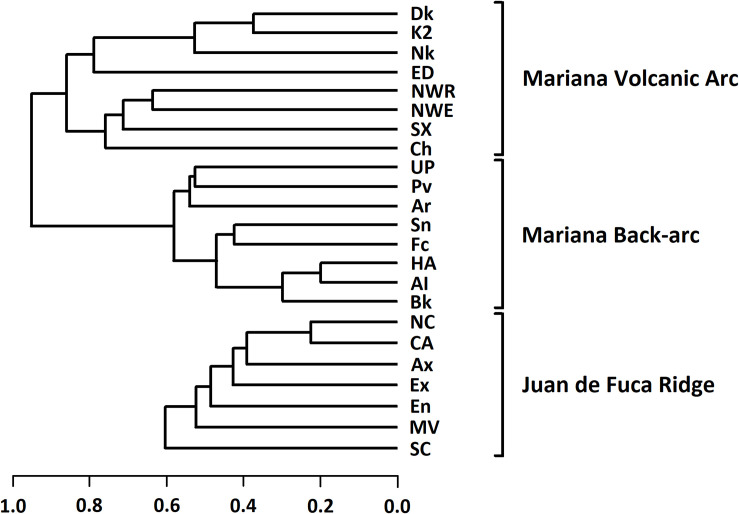
Dendrograms of hydrothermal vent macrofauna assemblages in the Mariana region and Juan de Fuca ridge. Dendrograms illustrating the relative dissimilarity (β-diversity using the Jaccard Index) of vent sites in each of the three vent systems investigated in this study. Site name abbreviations match those in Table 3. The Mariana systems are illustrated by a single dendrogram because they share six species. No species are shared across the North Pacific.

**Fig 3:**
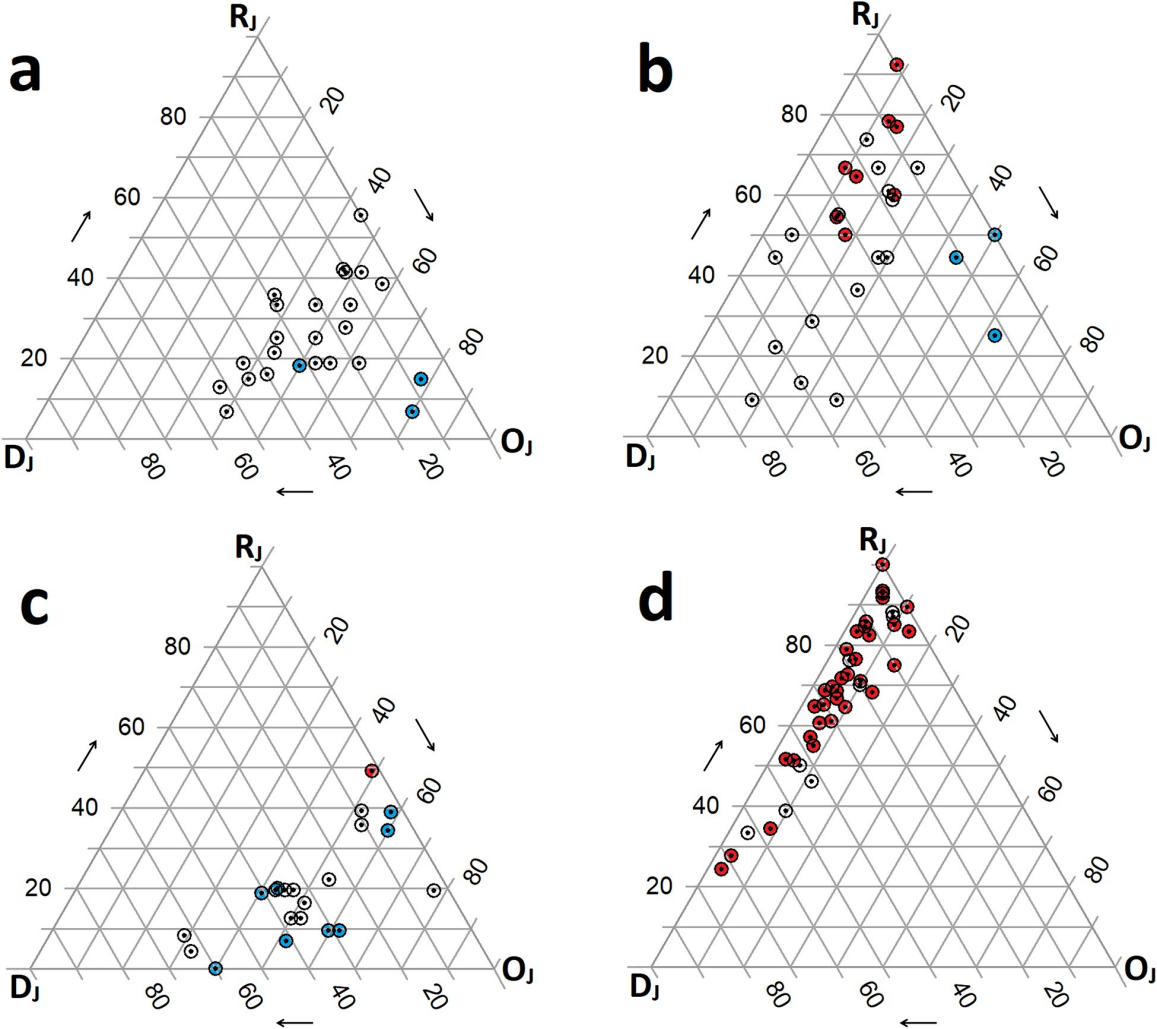
Simplex plots of hydrothermal vent macrofauna assemblages in the Mariana region and Juan de Fuca ridge. Simplex plots *sensu* Podani & Schmera [61] to illustrate the relative importance of additive pairwise pattern components (PPCs) *sensu* Schmera et al. [13] in diversity distributions. PPCs include species *overlap* (O_J_), relativized species *replacement* (R_J_) and relativized species *richness difference* (D_J_). Values are calculated for every site pair within each vent system. Red points indicate site pairs within which differences are significant, as calculated by the Raup-Crick Index. Blue points indicate pairs with significant similarity, while clear points represent pairs with no significant deviance from the expectation of random chance. Arrows show direction in which to read each axis. a) Mariana back-arc ridge system; b) Mariana volcanic arc system; c) Juan de Fuca/Explorer mid-ocean ridge system; d) Mariana region with back-arc and volcanic arc combined, illustrating between-system values only. Here, 16 between-system pairs that share no species lie at the top apex. Thus, the intersection of nestedness and β-diversity (I_J_) and the relative complement of nestedness in β-diversity (RC_J_) are used for calculation as O_J_ = 0 [13].

**Fig 4:**
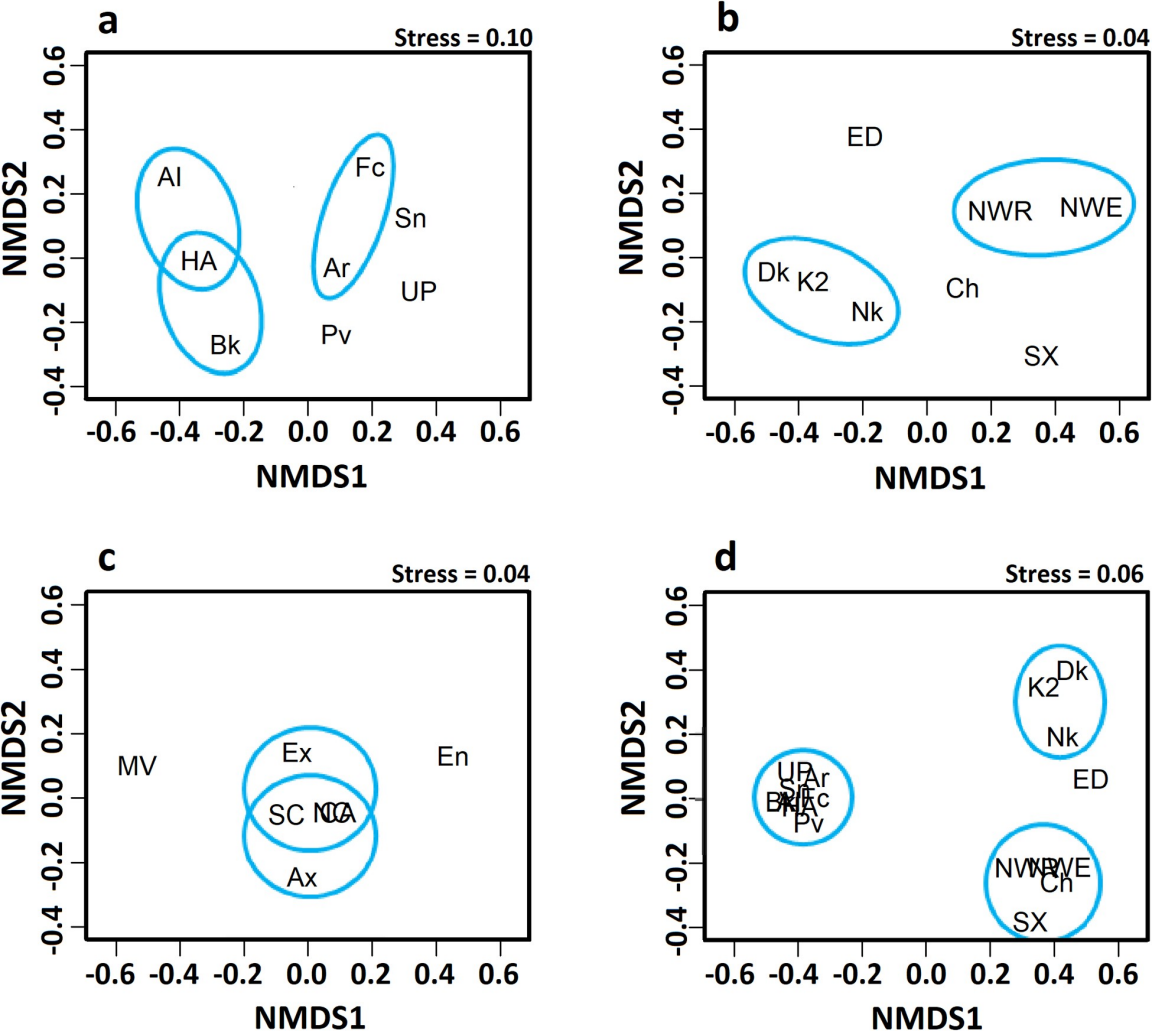
nMDS plots illustrating the β_RC_-diversity values of hydrothermal vent assemblages in the Mariana back-arc, volcanic arc and Juan de Fuca ridge. Non-metric multidimensional scaling (nMDS) plots illustrating the dissimilarity of vent sites in each system relative to the null expectation of random assembly (β_RC_-diversity). Site name abbreviations match those in Table 3. Blue ellipses represent the sites that are significantly more similar to each other (β_RC_-diversity). (a) Mariana back-arc ridge system; (b) Mariana volcanic arc system; (c) Juan de Fuca/Explorer mid-ocean ridge system; (d) Mariana region including the back-arc and volcanic arc.

The three northern sites (Alice Springs/Illium, Burke, Hafa Adai) form the tightest cluster (Fig 2). Comparisons between sites on the Mariana back-arc show a ‘complex’ pattern (*sensu* Schmera *et al*. [13]), in which all three PPCs are present in the site-by-species matrix, though not in all site pairs. The *overlap* PPC (O_J_) contributes most to the pattern with pairwise site values clustered in the lower-right corner of the simplex plot (Fig 3a). Pairwise β_J_ values range from 0.2 to 0.65 and indicate that, on average, these sites share ~49% of their species (Table 4). Perseverance is the only site with a significant local contribution to β-diversity (LCBD; p < 0.05). Pairwise N_J_ values are generally higher than the β_J_-diversity, ranging from 0.44 to 0.93. The average β_ratio_ for these sites is 0.49, indicating that the two components of β_J_-diversity (I_J_ and RC_J_) have nearly equal relative contributions overall. The average β_RC_ value is -0.33, and only ~11% (3/28) of these values fall beyond the 95% CIs (Table 4, S11 Table in S1 File). Hence, most paired sites are no more similar or different from each other than expected by random chance. Significant similarities are Hafa Adai with both Alice Springs/Illium and Burke, and Archaean with Forecast (Fig 4a).

**Table 4:** Species diversity measures for the three study vent systems.

Measures	Mariana back-arc	Mariana volcanic arc	Juan de Fuca ridge	Mariana Region (arc and back-arc)
β_J_	0.51 (0.53)	0.78 (0.82)**	0.50 (0.49)	0.81 (0.90)
N_J_	0.74 (0.77)	0.49 (0.48)**	0.80 (0.81)	0.45 (0.44)
I_J_	0.26 (0.27)	0.27 (0.23)	0.30 (0.33)	0.22 (0.19)
RC_J_	0.26 (0.23)	0.51 (0.52)**	0.20 (0.19)	0.58 (0.61)
β_ratio_	0.49 (0.54)	0.35 (0.3)	0.57 (0.66)	0.30 (0.27)
β_RC_	-0.33 (-0.34)	0.24 (0.65)**	-0.56 (-0.88)	0.23 (0.86)
% significant β_RC_ values	10.7	46.4	47.6	75

Abbreviations.

β_J_, pairwise Jaccard dissimilarity which is the inverse of the *overlap* (O_J_) PPC (β_J_ = 1 –O_J_).

N_J_, relativized nestedness *sensu* Schmera et al. [13].

I_J_, intersection of nestedness and β-diversity, which equals *richness difference* (D_J_) PPC when species *overlap* is > 0.

RC_J_, relative complement of nestedness in β-diversity (I_J_ + RC_J_ = β_J_); it is identical to the *replacement* (R_J_) PPC when species *overlap* is > 0.

β_ratio_, the proportional contribution of I_J_ in β_J_ *sensu* Dobrovolski et al. [67].

β_RC_, Raup-Crick index; significance is 95% CI on 9,999 permutations.

**Significant differences of β_J_, N_J_, D_J_/I_J_, R_J_/RC_J_, and β_RC_ values among the three systems; significance p < 0.05.

### Mariana volcanic arc

We identified 45 species, less than half of which have definitive species-level assignment (S5 Table in S1 File). For another eight, experts indicate ‘new species’ status, while the remainder require further assessment. Some better sampled sites returned fewer species than those with less sampling (e.g. 27 samples on NW Rota returned only nine species, while East Diamante had 16 species in nine samples). The expansive vent fields of Nikko and Daikoku need more investigation. The average number of species per site was 12.6 (Table 3). Only one species appears at all sites: the crab *Gandalfus yunohana*, while 47% (21/45) of species are known from only one site. Six species occur on both arc and back-arc: *Bathymodiolus septemdierum* (mussel), *L*. aff. *schrolli* MT (limpet), *B*. *marianus*, *Levensteiniella raisae* (both scaleworms), *D*. *marianaensis* and *P*. cf. *nassariaeformis* (both snails). While it is likely that further sampling will augment diversity and alter the specific results here, we highlight the overall pattern that is revealed in comparison to the back-arc that was sampled in a similar fashion dictated by the field conditions.

Dissimilarity among sites in the arc is generally greater than in the back-arc; the two systems form two high level clusters (Fig 2). The volcanic arc sites also present a ‘complex’ pattern; however, the *replacement* PPC (R_J_) contributes most to this pattern. Pairwise values on the simplex plot (Fig 3b) are less clustered than the back-arc (Fig 3a) and are more concentrated near the R_J_ apex. β_J_ values range from 0.38 to 0.94 and indicate that sites, on average, share 22% of species. LCBD is not significant for any sites. N_J_ values are notably lower than the β_J_-diversity, ranging from 0.08 to 0.91. Nestedness contributes less to β-diversity than its relative complement (Table 4), although it strongly influences some β-diversity values given that β_ratio_ values range from 0 to 0.89. Unlike the back-arc, the average β_RC_ value is positive (Table 4), and about 46% (13/28) of pairwise values fall beyond the 95% CIs. While three sites (Daikoku, Kasuga-2 and Nikko) and a pair (NW Eifuku and NW Rota2) are significantly more similar to each other (Fig 4b), most significant β_RC_ values are positive (S12 Table in S1 File).

### Juan de Fuca (JdF) ridge

This mid-ocean ridge system in the northeast Pacific is more speciose with 72 macrofaunal species identified–nearly all to species-level (S6 Table in S1 File). Average species per site is 30.4. The only sedimented site, Middle Valley, is one of the poorest sampled, yet returned the greatest species number (Table 3). At least half the species along the ridge are associated with the foundation species *Ridgeia piscesae* (tubeworm). Eleven species occurred at all seven sites, while 39% (28/71) are currently known only at one site, usually Middle Valley or Endeavour.

The JdF shows a cascade pattern of clusters with overall dissimilarity similar to the Mariana back-arc (Fig 2). Again, a ‘complex’ pattern is present, and, like the Mariana back-arc, O_J_ is the dominant PPC. Pairwise values also cluster near the O_J_ apex of the simplex plot (Fig 3c). β_J_ values range from 0.23 to 0.71 with ~50% of species shared on average (Table 4). LCBD is significant for South Cleft and Middle Valley sites (p < 0.001 and p < 0.05 respectively). N_J_ values generally exceed the β-diversity, ranging from 0.51 to 1 (perfect nestedness). As the average β_ratio_ is 0.57, nestedness contributes to the overall β-diversity more than its relative complement (Table 4). However, β_ratio_ values nearly span the full scale between 0.03 and 1. The average β_RC_ is -0.56, and ~48% (10/21) of the pairwise values fall beyond the 95% CIs, most of which are negative, although Endeavour and Middle Valley are significantly different (Fig 4c; S13 Table in S1 File).

### Between-system comparisons

β_J_ values differ among the three vent systems (F = 48.3; p = 0.001). Both the back-arc and JdF systems have significantly lower β_J_ values than the Mariana arc (both p < 0.001) (Table 4), although there is little difference between the Mariana back-arc and JdF Ridge (p = 0.98). The RC_J_ values of the arc are also significantly higher than those of the back-arc and JdF (both p < 0.001). However, I_J_ value differences are not significant among the three vent systems (F = 0.4; p = 0.68). Therefore, species overlap and replacement distinguish the volcanic arc from the two spreading ridges. Consistent with these results, β_RC_ values also differ among the three systems (F = 10.8; p = 0.001) with the arc having significantly higher β_RC_ values than the back-arc and JdF systems (p = 0.002 and p < 0.001, respectively), indicating that sites within the arc are more compositionally dissimilar relative to the random expectation compared to those of the other two systems. Although the proportion of significant β_RC_ values was notably higher in JdF than the back-arc, average values did not differ (p = 0.13) (Table 4).

In the combined Mariana region (volcanic arc and back-arc systems), R_J_ is the dominant influence on the ‘complex’, between-system pattern, while O_J_ had the smallest influence (Fig 3d). Between-system species replacement is high because only six of the total 75 species in the Mariana region are shared across the two systems. Overall, β_J_-diversity values between arc and back-arc sites range from 0.85 to 1. Compared to the lower among-system β_J_ values within these systems (Table 4), the average between-system β_J_ value is 0.95. Hafa Adai and NW Eifuku share the greatest proportion of species (15%) among between-system pairs, but 16 such pairs share no species. Overall, six sites have significant LCBDs, all of which are arc-hosted (Nikko, Daikoku, Chamorro, East Diamante, NW Rota and Seamount X; p < 0.05), thus reflecting the significantly greater dissimilarity among arc sites (Table 4).

Both the average between-system N_J_ and β_ratio_ values are low (Table 4), consistent with our observation of apparent species substitutions between the two systems. For example, bythograeid crabs occupy every Mariana site, but the species differs between systems. Similarly, several other related taxon pairs occupy similar niches on the arc and back-arc. The pairwise, between-system N_J_ (0.03 to 0.76) and β_ratio_ (0 to 0.75) values indicate that nestedness influences β-diversity in some between-system pairs more than the relative complement. However, large richness differences contribute most to the high between-system nestedness values.

In the combined species pool of the Mariana region, 75% (90/120) of the β_RC_ values are significant (S14 Table in S1 File). In this context, within-system β_RC_ values for the back-arc all exceed the negative CI, and 21% (6/28) of the within-system β_RC_ values for the volcanic arc are also more similar than random (Fig 4d) with one arc pair significantly different. In contrast, 86% (55/64) of the between-system β_RC_ values exceed the positive CI, emphasizing the differences between the arc and back-arc.

### Environmental drivers

For the Mariana back-arc, the non-linear correlation between habitat area and α-diversity lies on the threshold of significance (Kendall tau, α = 0.05, p = 0.05). No other environmental variables correlate with α-diversity. The dbMEM analyses also found no significant correlation between the abiotic variables and the β-diversity values.

## Discussion

We examined vent assemblages in three geotectonic settings: back-arc, volcanic arc and mid-ocean ridge. Differing diversity characteristics emerge among the systems, especially in the β-diversity partitions. Whittaker’s diversity index is similar between the back-arc and mid-ocean ridge systems, but much higher for the volcanic arc. In each, the relative contribution of each *pairwise pattern component* differs such that the β_ratio_ decreases from JdF to Mariana back-arc to volcanic arc reflecting the increasing role of the *replacement* PPC while *richness difference* plays a larger role in the JdF system. A study of the invertebrates of Finland streams in eight regions [84] resembles ours in both assemblage type and outcomes, but we find a greater range in mean *overlap* and *replacement* PPCs in only three equivalent ‘regions’.

Despite their proximity, Mariana volcanic arc and back-arc differ in both overall species composition (as noted by Kojima and Watanabe [44]) and spatial arrangement of diversity. β_J_-diversity (dissimilarity) is much higher on the arc compared to greater nestedness on the back-arc. Combining to a single Mariana region emphasizes the strong replacement component across systems. Phylogenetically related species occupy similar hydrothermal niches in the arc and back-arc; however, their origins likely reflect differing vicariant histories. The bythograeid crabs in arc and back-arc both have closer relatives in the south-west Pacific vent settings [85], and arc and back-arc *Alviniconcha* snails show markedly different phylogenetic divergence times [86]. While bathymetric differences between arc and back-arc sites may discourage faunal crossover, we note that *A*. *hessleri* larvae have been detected at 500 m depth [87]. It is likely that many vent species rise to the surface to feed, thus facilitating wider dispersion [88]. However, while surface dispersal occurs in the limpet *Shinkailepas myojinensis* on the Izu-Bonin arc [89] (contiguous with the Mariana arc), this species is replaced by others of the genus in each of the Mariana arc and back-arc sites. Thus, larval dispersability may not assure connectivity. Furthermore, Seamount X (arc) and Forecast (back-arc) are separated by only 20 km distance and less than 200 m depth, yet they share only 3 of the 33 species that we record at these sites. A study of ε-proteobacteria also noted the marked difference of microbial composition in Forecast fluids from those in arc fluids [52].

The differing interactions with underlying heat sources of the two systems affects the fundamental character of venting fluids [90], including compounds (e.g. CO_2_, CH_4_, H_2_S) that control toxicity and microbial productivity. The Mariana back-arc is characterized by extensive deposits of mineralized sulphides compared to few deposits on the arc; instead many sites are paved with elemental sulphur [51]. The differing substrata reflect excess sulphur (SO_2_ and SO_4_^-2^) in the arc volcanoes compared to the back-arc [39, 49, 51]. Abundant CO_2_ also reduces relative pH in arc fluids [91] compared to the back-arc. Detailed study that includes fluid constituents may point to factors that affect habitat suitability for colonizing fauna.

Among volcanic arc sites, the *replacement* PPC is also strong: over 46% (13/28) of site pair comparisons were significantly different from random (β_RC_). β_RC_-diversity values suggest strong deterministic processes in this system. The species replacement has no distinct pattern along the arc where similar variability is reflected in three microbial studies at many of the same sites: microbial mats [92], fluid bacteria [52] and fluid protists [93]. Several factors may affect these site to site differences. First, in a study of the northern Mariana and Izu-Bonin arcs, Watanabe *et al*. [41] find that water depth may explain the similarities among the three northern Mariana arc sites (<600 m), whereas nearby NW Eifuku fauna is distinctly different (1600 m). However, we note this latter site is most similar to shallow NW Rota (550 m) at the southern end of the arc, suggesting that depth may not be the single factor. Second, reduced connectivity may contribute to observed diversity patterns as currents flow across the arc, not along the structure [94], thus impeding larval exchange. Metaxas [95] finds larval behaviours that favour local retention on two arc volcanoes. Thirdly, in general, arc volcanoes can vary markedly as venting fluids have different rock and magma influences [90]. The three northern sites with similar faunae all have sulphur-rich fluids [51], whereas NW Eifuku and NW Rota emit CO_2_ dominated fluids [37].

Mariana back-arc β_J_-diversity is more similar to JdF Ridge, northeast Pacific, in that nestedness (*overlap* and *richness difference* PPCs together) is strong. On both systems, connectivity may be enhanced by the topographic structure of an axial valley that directs currents along-strike [96]. Some widespread species show little genetic structure among populations such as *R*. *piscesae* on JdF Ridge [97]. Nestedness on the back-arc may result from past exchanges among sites where extensive extinct chimneys once supported vigorous hydrothermal emissions. The waning sites, Perseverance and Urashima/Pika, have low α-diversity values, yet they show no significant (dis)similarity with other sites. Receding hydrothermal flow could generate a random sampling effect in these sites, similar to the effects of a bleaching event on coral species where α-diversity declined, but β_RC_ was unchanged [15]. As few back-arc site pairs were significant (β_RC_), stochastic processes dominate in community assembly. This back-arc is a young system (~3 MA) [98], which may influence gamma diversity compared to the 28 MA-old JdF [99] with greater species accumulation.

Nestedness in JdF reflects a ‘core species set’ stretching across the system. The high level of significant similarity of pairs among five sites suggests deterministic processes influencing community assembly. Three sites have experienced eruptions at least once in the past 30 years where disturbance-adapted species colonize rapidly [53, 100]. Eruptive disturbance could impose an ecological filter, reducing α-diversity as described by Chase *et al*. [15]. In contrast, Middle Valley and Endeavour host large sulphide deposits where venting has persisted over thousands of years [30]. However, the sediments of Middle Valley and bare rock of Endeavour support significantly different assemblages from each other, again implicating the role of substratum in diversity patterns. The Endeavour site has maintained activity with no known species loss over 35 years of observation. Stability and habitat complexity likely contribute to diversity accumulation: here, α-diversity is highest, and some eruption site species are replaced. Long-term stability is also a feature of vent sites in other locations [101]. Vent systems display a range of disturbance-stability reflecting the underlying dynamics of the hydrothermal heat source [32, 102], and our three systems show varying degrees of disturbance from eruptive activity. We visited Alice Springs, Mariana back-arc, 30 years after discovery [81], to find it virtually unchanged, but three arc sites have experienced eruptions since 2006 (e.g. NW Rota multiple times [29]).

The role of foundation species could affect differences in average α-diversity among systems. In the back-arc, the hairy snail *Alviniconcha hessleri* forms low mounds providing additional surface area for associated species while foundation species are absent from most arc sites. In contrast, the siboglinid tubeworm (*Ridgeia piscesae*) on JdF creates complex bush-like structures at every site that greatly expand the surface area accessing vent fluids and support a complex association of microbes and fauna [56]. For the Mariana back-arc, we found no environmental factors to explain differences in α-diversity and also no correlation between β-diversity and site distances, thus no support for the hypothesis of an along-strike faunal shift. Overall, the differences we observe in patterns of diversity distribution suggest that community assembly processes are not simple at hydrothermal vent ecosystems.

Our results are relevant to nations considering protection or mining in their waters and to the International Seabed Authority, the agency that will enable exploitation in seven (at present) high seas contracts. β-diversity analyses can help address criteria that identify significant areas for protection. Interactions among habitat suitability, geographic location and dispersal in the vent ecosystem influence the maintenance of metacommunities [103] in which species distribution reflects past or current connectivity. β-diversity patterns identify site linkages that can be tested with both genetic and network models to examine models of resilience to mining intrusions. For example, Suzuki *et al*. [104] use a dispersal model to predict very short recovery times for the Marianas, but it assumed incorrectly that any given species occurs at all sites on the volcanic arc and back-arc combined. Such models need grounding in distribution data to identify key nodes to maintain metacommunities. Dunn *et al*. [22] present a well-reasoned approach to placing broad conservation areas along the Mid-Atlantic Ridge where three ISA contracts exist. Citing the lack of available information on diversity distribution, they develop a framework based in habitat indicators and biodiversity drivers to address CBD criteria. A β-diversity analysis of the vent system here could test which siting scenarios would best support target criteria. Compiling data from expeditions of several nations and contractors remains a key requirement. Bonifácio *et al*. [105] demonstrate the role of such approaches, reporting high species replacement across mining contract areas in an abyssal manganese nodule province. Incorporating functional β-diversity into conservation planning would be highly effective [106, 107], but decisions on mining these deep-sea areas may not wait for acquisition of more than compositional data.

Most sites we examined are under some degree of protection (Mariana Trench Marine National Monument, Endeavour Hot Vents Marine Protected Area). Canada proposes to expand the Endeavour Hot Vents MPA to include Middle Valley and Explorer vent sites [108]. These three sites hold 89% of the known diversity in the region. While the remaining sites fall in international waters, protection of the three northern sites ensures habitat “safety” at large, relatively stable sites. As small geographic range is a superior predictor of extinction risk [109], conservation managers should consider that 75% of species occurring in our study area are endemic to the spreading ridges of the northeast Pacific. The results of our study provide strong support for the extended MPA and underscore the recommendation that siting MPAs consider β-diversity patterns [110]. The US Marine National Monument does not include the newly discovered sites on the Mariana back-arc. Here, Hafa Adai hosts the most extensive venting known in this system with a high faunal diversity. While Perseverance appears relatively depauperate, it is the only site with a significant local contribution to β-diversity due to a unique combination of species. We recommend inclusion of these sites in the Monument. Our study emphasizes just how limited distributions of these uniquely adapted species are; at least 60% of the Mariana back-arc species are known only from the very small habitat areas that we measured to a total of only about.02 km^2^.

The outcomes of β-diversity analyses provide decision support tools to achieve conservation objectives including species richness, site representativity, site replication and presence of species with restricted ranges [3]. Pairwise measures, such as PPCs, provide greater insight into diversity patterns than overall dissimilarity measures [111] by examining β-diversity partitions. The extent to which species among sites are similar, are replaced, or differ in species number, influences conservation choices. For example, the nested structure of birds on Amazonian cangas pointed to larger sites as targets for conservation with secondary sites to meet breeding needs [112]. We find that, on JdF Ridge, higher nestedness among sites suggests protection can focus on the more species-rich sites. The ratio of richness difference to replacement can identify the dominant PPC components in a system. With β_ratio_ < 0.5, a system such as the Mariana arc may require relatively more sites under protection to capture regional diversity [5]. Si *et al*. [9] recommend similar multi-site protection for lizard and bird communities on islands due to dominance of the replacement component. We supplemented the SET analysis with the Raup-Crick index (β_RC_) to identify pairs of sites that are less, or more, similar than random while controlling for richness difference. This index is often used to assess temporal changes in ecosystems under disturbance (e.g. [113]), thus may be applicable for long-term assessments of the impacts of seabed mining on diversity patterns. As β_RC_ also can determine whether community assembly over an ecosystem is deterministic, a search for possible drivers can begin. Knowledge of those drivers may help in deciding placement of conservation areas (such as vent sites that are sources for larvae for other sites [114]). Analyses that can document environmental drivers of observed patterns may simplify decision-making when inventories of species are not available. A predictive model, such as that developed for β-diversity in tree species [115], can identify environmental surrogates for conservation targets as the network of habitat sites expands. Another measure derived from β-diversity data is calculation of the “minimum set” of sites that include all species [116]. Our study finds 75% (6/8) of Mariana back-arc, 63% (5/8) of Mariana arc, and 71% (5/7) of JdF sites; these proportions are notably higher than a mean of 41% for 97 habitat island datasets [117]. Such values may be an intrinsic feature of hydrothermal vent systems which requires consideration in setting environmental objectives. Assessment of LCBD (local contribution to β-diversity) analyses provides more perspective to understand the differential roles of sites [66] and can complement minimum set analyses; they can also be partitioned to determine site contributions to both replacement and richness difference [12]. Hill *et al*. [118] demonstrate that replacement is dominant in diversity of urban ponds while identifying which ponds are most significant for conservation at the landscape scale. Overall, β-diversity approaches provide considerable information to reach a variety of conservation objectives in area selection, especially for networks of protected areas. Once established, β-diversity analysis can support monitoring approaches such as protected area effectiveness assessments (e.g. [119]) or to establish baselines for long-term trends within the designated area [120].

We examined only three vent systems, yet they show differing patterns. Unlike a similar study of three forests in which replacement dominates β-diversity [121], we find PPC contributions differ. Thus, vent systems cannot be treated as similar across regions nor can the same management approaches be applied. The differences in diversity distribution patterns suggest that community assembly processes vary at vent ecosystems. Further work can test the role of diversity drivers such as habitat size, habitat complexity, and stability. The best conservation approach for this special and rare ecosystem is to adopt the Vulnerable Marine Ecosystem designation from FAO [122] and place all active vents under protection from anthropogenic impacts, including mining for seafloor deposits. Regional β-diversity analyses for seamounts, another island-like ecosystem, would also support development of conservation plans in the face of fishing and planned mining pressures [123]. As pressures of human activities grow in the deep ocean, compilation and analysis of diversity data are critical to guide management decisions.

## Supporting information

S1 Fig Examples of hydrothermal habitats in Mariana back-arc (image credit Schmidt Ocean Institute). a) Low-lying habitat type with weak fluid delivery through cracks in the basalt at Alice Springs. Zonation patterns from higher to lower fluid exposure: *Alviniconcha hessleri* snails, *Neoverruca brachylepadoformis* barnacles and *Marianactis bythios* anemones in peripheral area. Image about 3 m across at bottom. b) A close up of the Sequoia chimney at Hafa Adai, illustrates vigorous fluid delivery as a black smoker. Bacterial mats on the left are grazed by alvinocaridid shrimp, hairy snails cluster in centre while limpets are abundant on the right. Image about 1.5 m across.
(TIF)

S1 File (DOCX)
