# Supplementary figures and images for: Beta diversity differs among hydrothermal vent systems: Implications for conservation
Source: PLoS One. 2021 Aug 26;16(8):e0256637. doi: 10.1371/journal.pone.0256637 (PMC8389485; doi:10.1371/journal.pone.0256637)

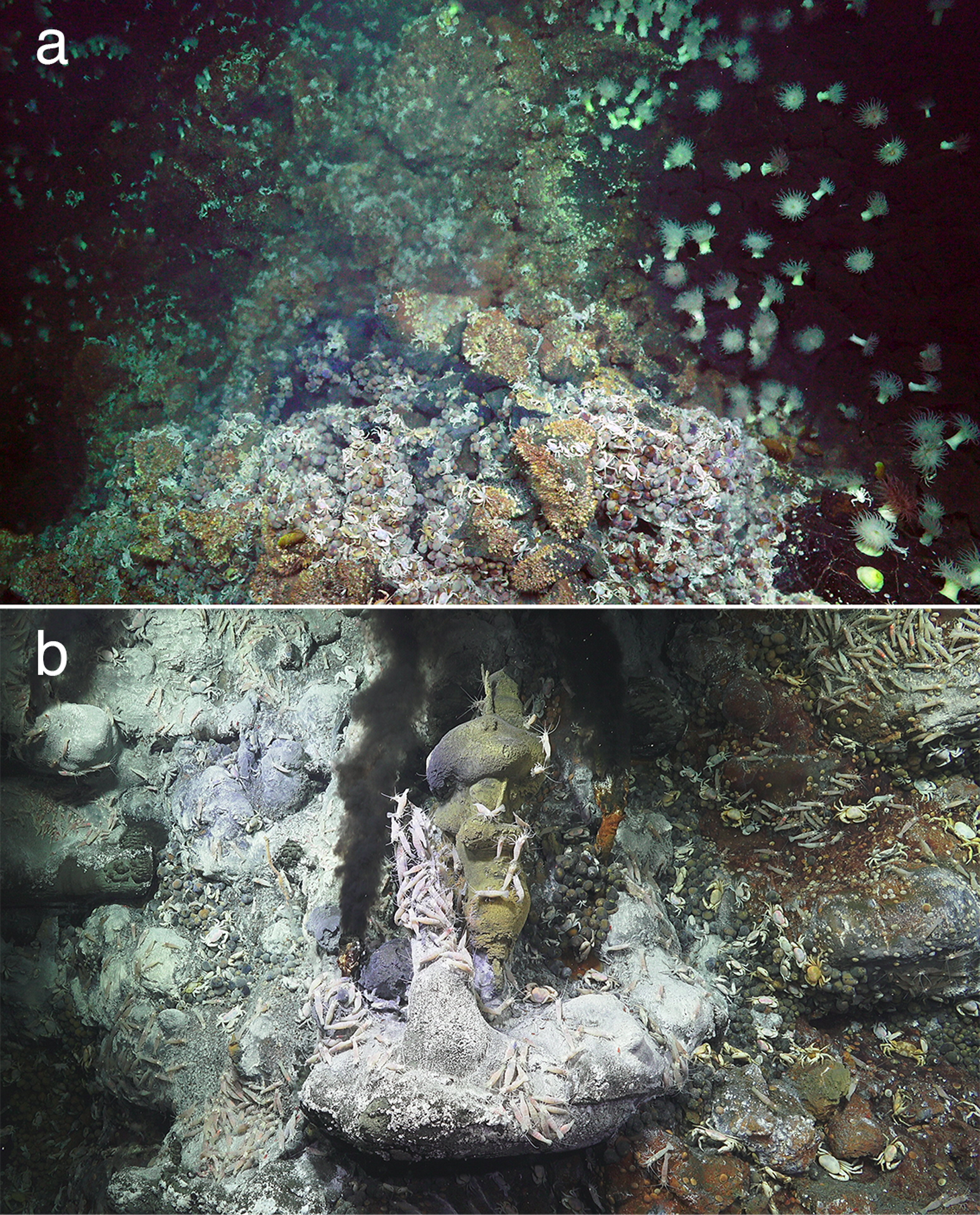

Supplement: S1 Fig — a) Low-lying habitat type with weak fluid delivery through cracks in the basalt at Alice Springs. Zonation patterns from higher to lower fluid exposure: Alviniconcha hessleri snails, Neoverruca brachylepadoformis barnacles and Marianactis bythios anemones in peripheral area. Image about 3 m across at bottom. b) A close up of the Sequoia chimney at Hafa Adai, illustrates vigorous fluid delivery as a black smoker. Bacterial mats on the left are grazed by alvinocaridid shrimp, hairy snails cluster in centre while limpets are abundant on the right. Image about 1.5 m across. (TIF) [file pone.0256637.s001.tif]
