# Supplementary material for: Beta diversity differs among hydrothermal vent systems: Implications for conservation
Source: PLoS One. 2021 Aug 26;16(8):e0256637. doi: 10.1371/journal.pone.0256637 (PMC8389485; doi:10.1371/journal.pone.0256637)
Supplement: S1 File — (DOCX) [file pone.0256637.s002.docx]

Beta diversity differs among hydrothermal vent systems:
implications for conservation

**S1 Text: General information on Mariana back-arc assemblages including new sites.**

Composition of the vent assemblages at the Mariana BA intergrades responding to the nature and volume of hydrothermal fluid delivery. Areas of sparse venting experience weak fluid delivery through cracks in low-lying basalt and sulphide substrata. At the three northernmost sites and at Snail, wafting fluids sponsor large expanses of actinarians (*Marianactis bythios*), zoantharians (*Epizoanthus* cf. nov. sp.) and squat lobsters (*Munidopsis* spp.) with occasional whelks (*Phymorhynchus wareni*) (S1a Fig). Nearer more focussed flow at all sites, squat lobsters and neoverrucid barnacles (*Neoverruca brachylepadoformis*) densities increase. Sparse mussels (*Bathymodiolus septemdierum*) and scalpellomorph barnacles (*Vulcanolepas* nov. sp.) may occur but never form dense assemblages. Rarely, white bacterial mats and blue folliculinid ciliates occur on the substratum. A transition through dense barnacles to piles of hairy snails (*Alviniconcha hessleri*) is rapid at sites with focussed venting through low-lying substrata; associated fauna is relatively diverse.

The Hafa Adai site has several tall black smoker chimneys rising 10 to 30 m in height. As the sulphide deposit is highly indurated, discrete exit points bathe parts of the upper chimney in fluid. The fauna forms a mosaic of patches with clusters of shrimp (*Rimicaris* spp.), limpets (*Shinkailepas* spp. and *Pseudorimula marianae*), hairy snails, other provannid snails (*Provanna nassariaeiformis* and *Desbruyeresia marianaensis*), and alvinellid polychaetes (*Paralvinella hessleri*) (S1b Fig). Archaean also has black smoker edifices, although smaller. Perseverance and Urashima/Pika sites are mostly inactive with extensive fields of non-venting chimneys. Here, excess dissolved iron is deposited as oxides coating the chimneys – particularly prevalent at the latter site. The few animals present are tightly clustered where the iron is reduced around trickling fluids.”

Tables provide information on each of the sites in our study.

S1 Table presents a summary of substrata and assemblages of the Mariana back-arc sites.

S2 Table presents summary features and references for the Mariana volcanic arc sites.

S3 **Table presents** summary features and references for the Juan de Fuca sites.

**S1 Table. Brief descriptions of the venting and faunal features observed at each of the Mariana back-arc vent sites.**

|  | **Alice Springs-Illium** | **Burke** | **Hafa Adai** | **Perseverance** | **Forecast** | **Snail** | **Archaean**† | **Urashima-Pika** |
| --- | --- | --- | --- | --- | --- | --- | --- | --- |
| **Coordinates** | 18˚12.7’N  144 ˚42.5’E | 18˚11.0’N 144˚43.2’E | 16˚57.7’N 144˚52.2’E | 15˚28.8’N 144˚30.5’E | 13˚2’N  143˚6’E | 12˚57.2’N 143˚37.2’E | 12˚56’N 143˚38’E | 12˚55.1’N 143˚38.9’E |
| **Vent features** | Clear, diffuse flow through basalt and sulphide rubble. No active chimney found. Many scattered vents in two fields | Clear, diffuse flow through basalt and sulphide rubble. Some inactive chimneys. | Black smokers on several tall chimneys and smaller pipes. Vents with diffuse flow through sulphide debris. Four areas of venting east-west. | Clear, diffuse flow through fallen sulphides and basalt cracks. Inactive chimneys with iron deposits; venting limited in extent. | Clear, diffuse flow through basalt and small sulphide mounds. | Clear, diffuse flow through basalt cracks and small sulphide mounds. Limited extent. | Large sulphide deposit with black smokers. | Inactive chimneys coated in thick iron deposits. Most fluid as trickles with rare jets. |
| **Assemblage character** | *Alviniconcha* surround outlets. Mosaic of species clusters over basalts with cnidarians extensive on periphery | *Alviniconcha* surround outlets; barnacles most abundant with cnidarians on periphery. | On chimneys, shrimp, limpets & crabs dominate. On lower mounds, dense piles of *Alviniconcha* with associates; barnacles and galatheids peripheral. | Sparse fauna on small outlets with shrimp, limpets and scattered *Alviniconcha*. Very low biomass. | *Alviniconcha* surround outlets with shrimp; galatheids on periphery. Low biomass | Small *Alviniconcha* piles with crabs; whelks and anemones surround. Low biomass | Shrimp and crabs dominate around outlets;  galatheids on periphery | Low density *Alviniconcha* and shrimp. No peripheral fauna. Very low biomass |

**S2 Table. Brief descriptions of the venting and faunal features observed at each of the Mariana volcanic arc vent sites.**

|  | **Nikko** | **Kasuga-2**  **(Dai-ni Kasuga)** | **NW Eifuku** | **Daikoku** | **Chamorro** | **East Diamante** | **NW Rota** | **Seamount X** |
| --- | --- | --- | --- | --- | --- | --- | --- | --- |
| **Coordinates** | 23˚05.0'N  142 ˚20.0'E | 21˚36.0'N 143˚37.2'E | 21˚29.1'N 144˚02.6'E | 21˚19.4'N 144˚11.6'E | 20˚48.6'N 144˚42.3'E | 15˚55.8'N 145˚40.2'E | 14˚36.1'N 144˚47.4'E | 13˚15.0'N 144˚01.2'E |
| **Distance to next seamount** | 211 | 46 | 24 | 78 | 550 | 176 | 170 |  |
| **Vent features** | Caldera with diffuse flows.  Liquid sulphur pools; gas emissions; substratum mostly sulphur | Limited diffuse venting through basalt & sulphur deposits and sediments. | High temperature vents near summit with liquid CO_2_ release; areas of iron oxide deposits over basalt. | Recent eruption with molten sulphur release; diffuse venting through sediments on flanks. | Limited low temperature venting through sulphide chimneys | High temperature smokers; diffuse venting on dacite slopes to photic zone. | Active volcano with recent eruptions; molten sulphur; diffuse venting.. | Limited low temperature vents through sulphur chimneys and debris. |
| **Assemblage character** | Extensive field of tubeworms with associated fauna in caldera; sulphur flows with abundant crabs, shrimp and tonguefish over large area. | Sparse populations of shrimp, tonguefish, snails and crab on sulphur debris & sediments. | Shrimp and limpets near sulphur chimneys; large mussel field over summit with associated fauna. | Crabs, shrimp and tonguefish abundant on sulphur flows; fish, snails and worms on/in sediments; sparse tubeworms. | *Alviniconcha* snails, polynoids on chimneys; peripheral galatheids & crabs. | *Alviniconcha* and crabs on chimneys; dense barnacle patches; limpets, snail, mats | Limited fauna; dense *Opaepele* shrimp near eruption pit; limpets, barnacles & crabs peripheral. | Scattered fauna; galatheids abundant; shrimp, limpets, few crabs; polychaetes in sediments |
| **Reports with assemblage /habitat information** | (1, 2) | (3) | (4) | (2, 5) |  | (6) | (4) |  |

**S3 Table. Brief descriptions of the venting and faunal features observed at each of the Juan de Fuca/Explorer Ridge vent sites.**

|  | **Explorer South** | **Middle Valley** | **Endeavour** | **CoAxial** | **Axial** | **North Cleft** | **South Cleft** |
| --- | --- | --- | --- | --- | --- | --- | --- |
| **Coordinates** | 49°45.0'N 130°16.0'W | 48°27.4'N 128°42.5'W | 47°56.9'N 129°5.9'W | 46°9.3'N 129°48.6'W | 45°55.6'N 129°58.8'W | 44° 8.8'N 130°12.0'W | 44°40.0'N 130°22.0'W |
| **Distance to next site south (km)** | 187 | 63 | 206 | 29 | 106 | 37 |  |
| **Vent features** | Massive sulphide deposit with active black smokers; diffuse venting through sulphide debris | Massive sulphide deposit mostly buried in turbidite sedments; small chimneys; diffuse venting through sediments. | Black smoker chimneys concentrated in five ventfields along strike; diffuse venting through basalt. | Small area with a few chimneys; mostly diffuse venting through recent basalts | Black smokers in two areas; extensive diffuse venting in caldera; recent eruptions. | A few black smokers; diffuse venting post eruption through basalts. | A few smokers and chimneys; iron oxides; vents in basalts and sediment drapes |
| **Assemblage character** | Alvinellids on smokers; tubeworm clusters with associated fauna; peripheral gastropods. | Tubeworm clusters with associated fauna; dense beds of clams with polychaetes; some seep fauna present. | Alvinellids on smokers; tubeworm clusters with associated fauna; peripheral gastropods. | Tubeworm clusters with associated fauna. | Alvinellids on smokers; tubeworm clusters with associated fauna; peripheral gastropods. | Alvinellids on smokers; tubeworm clusters with associated fauna; peripheral gastropods. | Alvinellids on smokers; tubeworm clusters with associated fauna; peripheral bivalves. |
| **Reports with assemblage /habitat information** | (7) | (8-10) | (11-13) | (14) | (15, 16) | (17, 18) |  |

**S4 Table. Mariana back-arc species presence data used for analyses.** Macrofauna data are compiled from our collections and from other species reports (see Kojima and Watanabe 2015, Cubelio et al. 2008; Chen & Watanabe, 2019) where we were confident the species differs from our collection. Additional presence records from these reports and from our assessment of JAMSTEC imagery are indicated in bold. Species identified from previous reports are indicated with ‘**’.

| **Class** | **Family** | **Species** | **Alice**  **Springs -**  **Illium** | **Burke** | **Hafa Adai** | **Perseve-rence** | **Fore-cast** | **Snail** | **Archaean** | **Urashima**  **Pika** |
| --- | --- | --- | --- | --- | --- | --- | --- | --- | --- | --- |
|  |  |  | **AI** | **Bk** | **HA** | **Pv** | **Fn** | **Sn** | **Ar** | **UP** |
| Anthozoa | Epizoanthidae | *Epizoanthus* cf. sp. nov. | 1 | 1 | 1 | 0 | 0 | **1** | 0 | 0 |
| Anthozoa | Kadosactinidae | *Marianactis bythios* | 1 | 1 | 1 | 0 | **1** | **1** | **1** | **1** |
| Bivalvia | Cuspidariidae | *Thermomya sulcata*** | 0 | 0 | 0 | 0 | 0 | **1** | 0 | 0 |
| Bivalvia | Mytilidae | *Bathymodiolus septemdierum* | 1 | 1 | 1 | 0 | **1** | 0 | **1** | 0 |
| Gastropoda | Anatomidae | *Anatoma* sp.** | 0 | 0 | 0 | 0 | 0 | 0 | **1** | 0 |
| Gastropoda | Lepetodrilidae | *Lepetodrilus* aff. *schrolli* MT | 1 | 1 | 1 | 1 | **1** | **1** | **1** | 0 |
| Gastropoda | Lepetodrilidae | *Pseudorimula marianae* | 1 | 1 | 1 | 0 | **1** | **1** | 0 | 0 |
| Gastropoda | Neomphalidae | *Symmetromphalus regularis* | **1** | 1 | 1 | 0 | **1** | 0 | 0 | 0 |
| Gastropoda | Pectinodontidae | *Bathyacmaea* sp. | 1 | 0 | 1 | 0 | 0 | 0 | 0 | 0 |
| Gastropoda | Peltospiridae | *Lirapex* sp.** | 0 | 0 | 0 | 0 | 0 | 0 | 0 | **1** |
| Gastropoda | Peltospiridae | *Pachydermia* cf. *sculpta*** | 0 | 0 | 0 | 0 | **1** | **1** | 0 | 0 |
| Gastropoda | Phenacolepadidae | *Shinkailepas* sp. nov. 4 | 1 | 1 | 1 | 1 | 0 | 0 | **1** | 0 |
| Gastropoda | Phenacolepadidae | *Shinkailepas* sp. nov. 6 | 0 | 0 | 0 | 0 | 0 | 0 | 0 | **1** |
| Gastropoda | Phenacolepadidae | *Shinkailepas* sp. nov. 7 | 0 | 0 | 0 | 0 | **1** | **1** | **1** | **1** |
| Gastropoda | Provannidae | *Alviniconcha hessleri* | 1 | 1 | 1 | 1 | **1** | **1** | **1** | **1** |
| Gastropoda | Provannidae | *Desbruyeresia chamorrensis* | 1 | 0 | 0 | 0 | 0 | 0 | 0 | 0 |
| Gastropoda | Provannidae | *Desbruyeresia marianaensis* | 1 | 1 | 1 | 1 | 1 | **1** | **1** | **1** |
| Gastropoda | Provannidae | *Provanna nassariaeformis* | 1 | 1 | 1 | 0 | 0 | **1** | 0 | 0 |
| Gastropoda | Raphitomidae | *Phymorhynchus wareni* | 1 | 1 | 1 | 0 | **1** | **1** | **1** | **1** |
| Gastropoda | N/A | *Ventsia* cf. *tricarinata*** | **1** | 0 | 0 | 0 | **1** | **1** | 0 | 0 |
| Hexanauplia | Eolepadidae | *Vulcanolepas* *verenae* | **1** | 0 | 1 | 0 | 0 | 0 | 0 | **1** |
| Hexanauplia | Neoverrucidae | *Neoverruca brachylepadoformis* | 1 | 1 | 1 | 1 | **1** | **1** | **1** | **1** |
| Malacostraca | Alvinocarididae | *Rimicaris falkorae* | 0 | 1 | 0 | 1 | 0 | 0 | 0 | 0 |
| Malacostraca | Alvinocarididae | *Rimicaris vandoverae* | 1 | 1 | 1 | 1 | 1 | **1** | **1** | **1** |
| Malacostraca | Alvinocarididae | *Rimicaris* cf. *variabilis* | 1 | 1 | 1 | 1 | 1 | **1** | 0 | **1** |
| Malacostraca | Bythograeidae | *Austinograea williamsi* | 1 | 1 | 1 | 1 | **1** | **1** | **1** | **1** |
| Malacostraca | Munidopsidae | *Munidopsis gracilis* | 0 | 0 | 0 | 0 | **1** | 0 | 0 | 0 |
| Malacostraca | Munidopsidae | *Munidopsis marianica* | 1 | 1 | 1 | 1 | **1** | **1** | **1** | **1** |
| Polychaeta | Alvinellidae | *Paralvinella hessleri* | **1** | 1 | 1 | 1 | 0 | **1** | 0 | **1** |
| Polychaeta | Ampharetidae | *Amphisamytha* sp. nov. 1 | 1 | 1 | 1 | 0 | 0 | **1** | 0 | 0 |
| Polychaeta | Hesionidae | *Sirsoe hessleri* | 1 | 0 | 0 | 0 | 0 | 0 | 0 | 0 |
| Polychaeta | Maldanidae | *Nicomache* sp.**** | **1** | 0 | 0 | 0 | 0 | **1** | 0 | 0 |
| Polychaeta | Polynoidae | *Branchinotogluma burkensis* | **1** | **1** | 1 | 0 | **1** | 0 | 0 | 0 |
| Polychaeta | Polynoidae | *Branchinotogluma marianus* | **1** | 0 | 1 | 0 | **1** | 0 | 0 | 0 |
| Polychaeta | Polynoidae | *Lepidonotopodium minutum* | 1 | 1 | 1 | 0 | 0 | 0 | 0 | 0 |
| Polychaeta | Polynoidae | *Levensteiniella raisae* | 1 | 0 | 1 | 1 | **1** | **1** | 0 | **1** |
| Polychaeta | Spionidae | *Laonice* sp. nov. | 1 | 0 | 0 | 1 | 0 | 0 | 0 | 0 |
| Pycnogonida | Ammotheidae | *Sericosura cochleifovea* | 0 | 1 | 0 | 0 | 0 | 0 | 0 | 0 |
| Solenogastres | Simrothiellidae | *Helicoradomenia* sp. nov. | 0 | 1 | 1 | 0 | 0 | 0 | 0 | 0 |

**S5 Table.** **Presence data used for analyses for Mariana volcanic arc macrofauna.** The second row shows the abbreviations used for figures in the main text. Additional presence records from Watanabe et al. (2019) are bolded. The last column indicates if a barcode sequence is deposited for the species (COI); “morphology” means an identity comparing to literature descriptions, and an expert name is given in cases where we sought additional input.

| **Class** | **Family** | **Species** | **Nikko** | **Kasu-ga-2** | **NW Eifuku** | **Daikoku** | **Chamor-ro** | **East Diam-ante** | **NW Rota** | **Sea-mount X** | **Confirmation** |
| --- | --- | --- | --- | --- | --- | --- | --- | --- | --- | --- | --- |
|  |  |  | **Nk** | **K2** | **NWE** | **Dk** | **Ch** | **ED** | **NWR** | **SX** |  |
| Actinopterygii | Cynoglossidae | *Symphurus thermophilus* | 1 | 1 | 0 | 1 | 0 | 0 | 0 | 0 | COI |
| Anthozoa | Hormathiidae | Unk. sp. | 0 | 0 | 1 | 1 | 0 | 1 | 0 | 0 | J. Reimer |
| Bivalvia | Mytilidae | *Bathymodiolus septemdierum* | 1 | 1 | 1 | 0 | 0 | 0 | 0 | 0 | COI |
| Bivalvia | Mytilidae | *Gigantidas horikoshii* | 1 | 0 | 0 | 0 | 0 | 0 | 0 | 0 | morphology |
| Demospongiae | Chalinidae | *Haliclona* sp. | 0 | 0 | 0 | 0 | 0 | 1 | 0 | 0 | H Reiswig |
| Demospongiae | Suberitidae | *Pseudosuberites* sp. | 0 | 0 | 0 | 0 | 0 | 1 | 0 | 0 | H Reiswig |
| Gastropoda | Calliostomatidae | nr. *Calliostoma* sp. | 0 | 0 | 0 | 0 | 0 | 1 | 0 | 0 | morphology |
| Gastropoda | Colloniidae | *Homalopoma bicolor* | 0 | **1** | 0 | **1** | 0 | 0 | 0 | 0 | morphology |
| Gastropoda | Elachisinidae | *Laeviphitus* cf. *japonicus* | 0 | 1 | 1 | 1 | 0 | 1 | 0 | 1 | A. Warèn;  possibly new |
| Gastropoda | Lepetodrilidae | *Lepetodrilus* aff. *schrolli* MT | 0 | 0 | 1 | 0 | 0 | 0 | 1 | 1 | COI |
| Gastropoda | Lepetodrilidae | *Lepetodrilus* sp. nov. | 0 | 0 | 1 | 0 | 0 | 1 | 0 | 0 | A. Warèn |
| Gastropoda | Lepetodrilidae | *Pseudorimula* sp. nov. | 0 | 0 | 1 | 0 | 0 | 0 | 0 | 0 | A. Warèn |
| Gastropoda | Mangeliidae | *Oenopota ogasawarana* | 1 | **1** | 0 | 1 | 0 | 0 | 0 | 0 | A. Warèn |
| Gastropoda | Phenacolepadidae | *Shinkailepas kaikatensis* | 0 | 0 | 0 | 0 | 0 | 1 | 0 | 0 | Y. Kano |
| Gastropoda | Phenacolepadidae | *Shinkailepas* sp. nov. | 0 | 0 | 1 | 0 | 0 | 0 | 1 | 0 | Y. Kano |
| Gastropoda | Provannidae | *Alviniconcha* *adamantis* | 0 | 0 | 0 | 0 | 1 | 1 | 0 | 0 | COI |
| Gastropoda | Provannidae | *Desbruyeresia* cf. *marianaensis* | 0 | 1 | 1 | 0 | 0 | 1 | 0 | 1 | C. Chen |
| Gastropoda | Provannidae | *Provanna* aff. *fenestrata* | 0 | 0 | 1 | 0 | 0 | 0 | 0 | 0 | A. Warèn  and C. Chen |
| Gastropoda | Provannidae | *Provanna* cf. *nassariaeformis* | 0 | 0 | **1** | 0 | 0 | 0 | 0 | 0 | C. Chen |
| Gastropoda | Skeneidae | Unk. sp. | 0 | 0 | 0 | 0 | 0 | 0 | 0 | 1 | morphology |
| Gastropoda | Trochidae | nr. *Monodonta* sp. | 0 | 0 | 0 | 0 | 0 | 1 | 0 | 0 | morphology |
| Hexanauplia | Neoverrucidae | *Neoverruca intermedia* | 1 | 1 | 0 | 1 | 0 | 1 | 1 | 0 | H. Watanabe |
| Malacostraca | Alvinocarididae | *Alvinocaris marimonte* | 0 | 0 | 1 | 0 | 0 | 0 | 1 | 1 | COI |
| Malacostraca | Alvinocarididae | *Opaepele loihi* | 1 | 0 | 1 | 0 | 1 | 0 | 1 | 1 | COI |
| Malacostraca | Alvinocarididae | *Alvinocaris* sp. nov. | 0 | 0 | 0 | 0 | 0 | 0 | 0 | 1 | COI |
| Malacostraca | Bythograeidae | *Gandalfus yunohana* | 1 | 1 | **1** | 1 | 1 | 1 | 1 | 1 | morphology |
| Malacostraca | Galatheidae | *Munidopsis myojinensis* | 0 | 0 | 1 | 0 | 1 | 0 | 0 | 1 | video only |
| Malacostraca | Palaemonidae | *Periclimenes cannaphilus* | 1 | 1 | 0 | 1 | 0 | 0 | 0 | 0 | morphology |
| Malacostraca | Parapaguridae | *Paragiopagurus ventilatus* | 1 | 0 | 1 | 1 | 0 | 1 | 0 | 0 | morphology |
| Malacostraca | Xenograpsidae | *Xenograpsus novaeinsularis* | 0 | 1 | 0 | 1 | 0 | 1 | 0 | 0 | morphology |
| Malacostraca  (Brachyura) | Unknown | Unk. sp. | 0 | 0 | 0 | 0 | 0 | 1 | 0 | 0 | morphology |
| Polychaeta | Ampharetidae | *Amphisamytha* sp. nov. 2 | 0 | 0 | 1 | 0 | 0 | 0 | 0 | 0 | G. Rouse  and M. Eilertsen |
| Polychaeta | Capitellidae | *Capitella* sp. | 0 | 0 | 0 | 0 | 0 | 0 | 0 | 1 | J. Blake &  N. Maciolek |
| Polychaeta | Hesionidae | sp. 1 | 0 | 0 | 1 | 0 | 0 | 0 | 0 | 0 | morphology |
| Polychaeta | Hesionidae | sp. 2 | 0 | 0 | 1 | 0 | 0 | 0 | 1 | 0 | morphology |
| Polychaeta | Hesionidae | sp. 4 | 0 | 0 | 0 | 0 | 0 | 0 | 0 | 1 | morphology |
| Polychaeta | Hesionidae | sp. 5 | 0 | 0 | 0 | 0 | 0 | 0 | 0 | 1 | morphology |
| Polychaeta | Polynoidae | *Branchinotogluma marianus* | 1 | 0 | 1 | 0 | 1 | 1 | 1 | 1 | S. Hourdez |
| Polychaeta | Polynoidae | *Branchinotogluma* sp. nov. | 0 | 0 | 1 | 0 | 0 | 0 | 0 | 0 | S. Hourdez |
| Polychaeta | Polynoidae | *Levensteiniella raisae* | 0 | 0 | 1 | 0 | 0 | 0 | 1 | 0 | S. Hourdez |
| Polychaeta | Siboglinidae | *Lamellibrachia satsuma* | 1 | 1 | 0 | 1 | 0 | 0 | 0 | 0 | morphology |
| Polychaeta | Spionidae | *Malacoceros* sp. | 0 | 0 | 0 | 1 | 0 | 0 | 0 | 0 | J. Blake &  N. Maciolek |
| Polychaeta | Spionidae | *Prionospio* sp. | 0 | 0 | 0 | 0 | 0 | 0 | 0 | 1 | J. Blake &  N. Maciolek |
| Polyplacophora | Protochitonidae | *Deshayesiella sirenkoi* | **1** | **1** | 0 | **1** | 0 | 0 | 0 | 0 | morphology |
| Solenogastres | Simrothiellidae | *Helicoradomenia* sp. nov. | 0 | 0 | 0 | 1 | 0 | 0 | 0 | 0 | morphology |

**S6 Table. Presence data used for analyses of Juan de Fuca Ridge macrofauna.** The second row shows abbreviations used for figures in the main text.

| **Class** | **Family** | **Species** | **Explorer** | **Middle**  **Valley** | **Endeavour** | **Co-Axial** | **Axial** | **North Cleft** | **South**  **Cleft** |
| --- | --- | --- | --- | --- | --- | --- | --- | --- | --- |
|  |  |  | **Ex** | **MV** | **En** | **CA** | **Ax** | **NC** | **SC** |
| Actinopterygii | Zoarcidae | *Pachycara gymninium* | 0 | 0 | 1 | 0 | 1 | 0 | 0 |
| Asteroidea | Xyloplactidae | *Xyloplax* sp. nov. | 0 | 0 | 1 | 0 | 0 | 0 | 0 |
| Bivalvia | Mytilidae | *Benthomodiolus erebus* | 0 | 1 | 1 | 0 | 0 | 0 | 0 |
| Bivalvia | Mytilidae | *Idas washingtonia* | 0 | 1 | 0 | 0 | 1 | 0 | 0 |
| Bivalvia | Solemyidae | *Acharax johnsoni* | 0 | 1 | 0 | 0 | 0 | 0 | 0 |
| Bivalvia | Vesicomyidae | *Calyptogena starobogatovi* | 0 | 1 | 1 | 0 | 1 | 0 | 1 |
| Bivalvia | Vesicomyidae | *Vesicomya diagonalis* | 0 | 1 | 0 | 0 | 0 | 0 | 0 |
| Gastropoda | Buccinidae | *Buccinum thermophilum* | 0 | ? | 1 | 0 | 0 | 0 | 0 |
| Gastropoda | Buccinidae | *Buccinum viridum* | 1 | 1 | 0 | 0 | 0 | 0 | 0 |
| Gastropoda | Cancellariidae | *Admete verenae* | 0 | 0 | 1 | 0 | 0 | 0 | 0 |
| Gastropoda | Cerithiopsidae | *Speculator cariosus* | 1 | 0 | 0 | 0 | 0 | 0 | 0 |
| Gastropoda | Cocculinidae | *Pyropelta* cf. *craigsmithi* | 0 | 1 | 0 | 0 | 0 | 0 | 0 |
| Gastropoda | Fissurellidae | *Puncturella* sp. nov. | 0 | 0 | 0 | 0 | 1 | 0 | 0 |
| Gastropoda | Fissurelllidae | *Cornisepta verenae* | 0 | 0 | 0 | 0 | 1 | 0 | 0 |
| Gastropoda | Hyalogyrinidae | *Hyalogyrina globularis* | 1 | 1 | 0 | 0 | 1 | 0 | 0 |
| Gastropoda | Lepetodrilidae | *Clypeosectus curvus* | 1 | 1 | 1 | 1 | 1 | 1 | 0 |
| Gastropoda | Lepetodrilidae | *Lepetodrilus corrugatus* | 0 | 1 | 0 | 0 | 0 | 0 | 0 |
| Gastropoda | Lepetodrilidae | *Lepetodrilus fucensis* | 1 | 1 | 1 | 1 | 1 | 1 | 1 |
| Gastropoda | Melanodrymiidae | *Melanodrymia brightae* | 1 | 0 | 1 | 0 | 0 | 0 | 0 |
| Gastropoda | Neolepetopsidae | *Paralepetopsis tunnicliffae* | 0 | 1 | 0 | 0 | 0 | 0 | 0 |
| Gastropoda | Neomphalidae | *Lacunoides vitreus* | 0 | 0 | 0 | 0 | 1 | 0 | 0 |
| Gastropoda | Newtoniellidae | *Eumetula* sp. nov. | 1 | 0 | 0 | 0 | 0 | 0 | 0 |
| Gastropoda | Peltospiridae | *Depressigyra globulus* | 1 | 1 | 1 | 1 | 1 | 1 | 1 |
| Gastropoda | Provannidae | *Provanna variabilis* | 1 | 1 | 1 | 1 | 1 | 1 | 0 |
| Gastropoda | Pyropeltidae | *Pyropelta musaica* | 0 | 1 | 0 | 0 | 1 | 0 | 0 |
| Gastropoda | Sutilizonidae | *Sutilizona tunnicliffae* | 0 | 0 | 1 | 0 | 0 | 0 | 0 |
| Gastropoda | Sutilizonidae | *Temnocinclis euripes* | 1 | 0 | 1 | 1 | 1 | 1 | 1 |
| Gastropoda | Skeneidae | *Fucaria striata* | 0 | 1 | 1 | 0 | 0 | 0 | 0 |
| Hoplonemertea | Emplectonematidae | *Thermanemertes valens* | 0 | 0 | 0 | 1 | 0 | 1 | 0 |
| Malacostraca | Callopiidae | *Oradarea* nr. *longimana* | 0 | 0 | 1 | 0 | 0 | 0 | 0 |
| Malacostraca | Galatheidae | *Munidopsis alvisca* | 1 | 1 | 1 | 1 | 1 | 0 | 0 |
| Malacostraca | Oregoniidae | *Macroregonia macrochira* | 1 | 1 | 1 | 1 | 1 | 1 | 1 |
| Malacostraca | Pardaliscidae | *Pardalisca endeavouri* | 0 | 0 | 1 | 0 | 0 | 0 | 0 |
| Malacostraca | Sebidae | *Seba profunda* | 0 | 0 | 1 | 0 | 0 | 0 | 0 |
| Malacostraca | Cytheruridae | *Xylocythere sarrazinae* | 0 | 1 | 1 | 0 | 1 | 1 | 0 |
| Malacostraca | Philomedidae | *Euphilomedes climax* | 1 | 1 | 1 | 1 | 1 | 1 | 0 |
| Polychaeta | Alvinellidae | *Paralvinella dela* | 0 | 1 | 1 | 0 | 1 | 1 | 0 |
| Polychaeta | Alvinellidae | *Paralvinella palmiformis* | 1 | 1 | 1 | 1 | 1 | 1 | 1 |
| Polychaeta | Alvinellidae | *Paralvinella pandorae* | 1 | 1 | 1 | 1 | 1 | 1 | 1 |
| Polychaeta | Alvinellidae | *Paralvinella sulfincola* | 1 | 1 | 1 | 1 | 1 | 1 | 1 |
| Polychaeta | Ampharetidae | *Amphisamytha carldarei* | 1 | 1 | 1 | 1 | 1 | 1 | 1 |
| Polychaeta | Capitellidae | *Capitella* nr. *capitata* | 0 | 1 | 0 | 0 | 0 | 0 | 0 |
| Polychaeta | Cirratulidae | *Chaetozone* sp. nov. | 0 | 1 | 0 | 0 | 0 | 0 | 0 |
| Polychaeta | Dorvilleidae | *Ophryotrocha globopalpata* | 0 | 1 | 1 | 1 | 1 | 1 | 1 |
| Polychaeta | Dorvilleidae | *Parougia wolfi* | 0 | 0 | 0 | 0 | 1 | 1 | 1 |
| Polychaeta | Hesionidae | *Amphiduropsis axialensis* | 0 | 1 | 1 | 1 | 1 | 0 | 0 |
| Polychaeta | Hesionidae | *Hesiodeira glabra* | 0 | 0 | 0 | 1 | 1 | 1 | 0 |
| Polychaeta | Hesionidae | *Hesiospina legendrei* | 0 | 0 | 1 | 0 | 0 | 0 | 0 |
| Polychaeta | Hesionidae | *Hesiospina vestimentifera* | 1 | 0 | 1 | 1 | 1 | 1 | 0 |
| Polychaeta | Lacydoniidae | *Lacydonia* sp. nov*.* | 0 | 1 | 0 | 0 | 0 | 0 | 0 |
| Polychaeta | Maldanidae | *Nicomache* cf. *arwidssoni* | 0 | 1 | 0 | 0 | 0 | 0 | 0 |
| Polychaeta | Maldanidae | *Nicomache venticola* | 1 | 1 | 1 | 0 | 1 | 1 | 1 |
| Polychaeta | Nereidae | *Nereis piscesae* | 0 | 1 | 0 | 0 | 1 | 0 | 0 |
| Polychaeta | Orbiniidae | *Berkeleyia lelievrei* | 0 | 0 | 1 | 0 | 0 | 0 | 0 |
| Polychaeta | Orbiniidae | *Leitoscoloplos pachybranchiatus* | 0 | 1 | 1 | 1 | 0 | 1 | 0 |
| Polychaeta | Orbiniidae | *Orbiniella hobsonae* | 1 | 1 | 1 | 0 | 1 | 0 | 0 |
| Polychaeta | Phyllodocidae | *Protomystides verenae* | 1 | 1 | 1 | 1 | 1 | 1 | 0 |
| Polychaeta | Polynoidae | *Branchinotogluma tunnicliffeae* | 1 | 1 | 1 | 1 | 1 | 1 | 1 |
| Polychaeta | Polynoidae | *Harmothoe globosa* | 0 | 0 | 0 | 1 | 1 | 0 | 0 |
| Polychaeta | Polynoidae | *Lepidonotopodium piscesae* | 1 | 1 | 1 | 1 | 1 | 1 | 1 |
| Polychaeta | Polynoidae | *Levensteiniella kincaidi* | 1 | 1 | 1 | 1 | 1 | 1 | 0 |
| Polychaeta | Scalibregmatidae | *Axiokebuita minuta* | 0 | 0 | 0 | 0 | 1 | 0 | 0 |
| Polychaeta | Siboglinidae | *Lamellibrachia barhami* | 0 | 1 | 0 | 0 | 0 | 0 | 0 |
| Polychaeta | Siboglinidae | *Ridgeia piscesae* | 1 | 1 | 1 | 1 | 1 | 1 | 1 |
| Polychaeta | Spionidae | *Lindaspio southwardorum* | 0 | 1 | 0 | 0 | 0 | 0 | 0 |
| Polychaeta | Spionidae | *Prionospio* sp*.* | 1 | 1 | 1 | 1 | 1 | 1 | 0 |
| Polychaeta | Syllidae | *Sphaerosyllis ridgensis* | 1 | 1 | 1 | 0 | 0 | 0 | 0 |
| Pycnogonida | Ammotheidae | *Sericosura dissita* | 0 | 0 | 1 | 0 | 0 | 0 | 0 |
| Pycnogonida | Ammotheidae | *Sericosura venticola* | 0 | 0 | 1 | 0 | 0 | 0 | 0 |
| Pycnogonida | Ammotheidae | *Sericosura verenae* | 1 | 1 | 1 | 1 | 1 | 1 | 1 |
| Solenogastres | Simrothiellidae | *Helicoradomenia juani* | 1 | 1 | 1 | 1 | 1 | 1 | 0 |

**S7 Table. The *pairwise pattern component* (PPC) values sensu Schmera et al. (2020) for the Mariana back-arc vent sites based on the Jaccard-family of indices.** The PPCs include species *overlap* (O_J_), relativized species *replacement* (R_J_) and relativized species *richness difference* (D_J_). These values are illustrated in Fig 3A.

|  | **Alice Springs-Illium** | **Burke** | **Hafa Adai** | **Perseverance** | **Forecast** | **Snail** | **Archaean** |
| --- | --- | --- | --- | --- | --- | --- | --- |
| **Burke** | O_J_ = 0.63  R_J_ = 0.19  D_J_ = 0.19 |  |  |  |  |  |  |
| **Hafa Adai** | O_J_ = 0.8  R_J_ =0.07  D_J_ = 0.13 | O_J_ = 0.78  R_J_ = 0.15  D_J_ = 0.08 |  |  |  |  |  |
| **Perseverance** | O_J_ = 0.4  R_J_ = 0.07  D_J_ = 0.53 | O_J_ = 0.44  R_J_ = 0.16  D_J_ = 0.4 | O_J_ = 0.41  R_J_ = 0.15  D_J_ = 0.44 |  |  |  |  |
| **Forecast** | O_J_ = 0.53  R_J_ = 0.19  D_J_ = 0.28 | O_J_ = 0.48  R_J_ = 0.41  D_J_ = 0.1 | O_J_ = 0.55  R_J_ = 0.28  D_J_ = 0.17 | O_J_ = 0.38  R_J_ = 0.33  D_J_ = 0.29 |  |  |  |
| **Snail** | O_J_ = 0.56  R_J_ = 0.19  D_J_ = 0.25 | O_J_ = 0.52  R_J_ = 0.41  D_J_ = 0.07 | O_J_ = 0.53  R_J_ = 0.33  D_J_ = 0.13 | O_J_ = 0.42  R_J_ = 0.25  D_J_ = 0.33 | O_J_ = 0.58  R_J_ = 0.38  D_J_ = 0.04 |  |  |
| **Archaean** | O_J_ = 0.35  R_J_ = 0.13  D_J_ = 0.52 | O_J_ = 0.44  R_J_ = 0.16  D_J_ = 0.4 | O_J_ = 0.41  R_J_ = 0.15  D_J_ = 0.44 | O_J_ = 0.44  R_J_ = 0.56  D_J_ = 0 | O_J_ = 0.5  R_J_ = 0.18  D_J_ = 0.32 | O_J_ = 0.42  R_J_ = 0.25  D_J_ = 0.33 |  |
| **Urashima-Pika** | O_J_ = 0.38  R_J_ = 0.19  D_J_ = 0.44 | O_J_ = 0.36  R_J_ = 0.36  D_J_ = 0.29 | O_J_ = 0.43  R_J_ = 0.21  D_J_ = 0.36 | O_J_ = 0.47  R_J_ = 0.42  D_J_ = 0.11 | O_J_ = 0.46  R_J_ = 0.33  D_J_ = 0.21 | O_J_ = 0.5  R_J_ = 0.25  D_J_ = 0.25 | O_J_ = 0.47  R_J_ = 0.42  D_J_ = 0.11 |

**S8 Table.** **The *pairwise pattern component* (PPC) values sensu Schmera et al. (2020) for the Mariana volcanic arc vent sites based on the Jaccard-family of indices.** The PPCs include species *overlap* (O_J_), relativized species *replacement* (R_J_) and relativized species *richness difference* (D_J_). These values are illustrated in Fig 3B

|  | **Nikko** | **Kasuga-2** | **NW Eifuku** | **Daikoku** | **Chamorro** | **E Diamante** | **NW Rota** |
| --- | --- | --- | --- | --- | --- | --- | --- |
| **Kasuga-2** | O_J_ = 0.5  R_J_ = 0.5  D_J_ = 0 |  |  |  |  |  |  |
| **NW Eifuku** | O_J_ = 0.18  R_J_ = 0.5  D_J_ = 0.32 | O_J_ = 0.14  R_J_ = 0.55  D_J_ = 0.31 |  |  |  |  |  |
| **Daikoku** | O_J_ = 0.44  R_J_ = 0.44  D_J_ = 0.11 | O_J_ = 0.63  R_J_ = 0.25  D_J_ = 0.13 | O_J_ = 0.13  R_J_ = 0.65  D_J_ = 0.23 |  |  |  |  |
| **Chamorro** | O_J_ = 0.21  R_J_ = 0.29  D_J_ = 0.5 | O_J_ = 0.06  R_J_ = 0.5  D_J_ = 0.44 | O_J_ = 0.18  R_J_ = 0.09  D_J_ = 0.73 | O_J_ = 0.06  R_J_ = 0.44  D_J_ = 0.5 |  |  |  |
| **E Diamante** | O_J_ = 0.17  R_J_ = 0.67  D_J_ = 0.17 | O_J_ = 0.22  R_J_ = 0.61  D_J_ = 0.17 | O_J_ = 0.23  R_J_ =0.6  D_J_ =0.17 | O_J_ = 0.25  R_J_ = 0.67  D_J_ = 0.08 | O_J_ = 0.17  R_J_ = 0.22  D_J_ = 0.61 |  |  |
| **NW Rota** | O_J_ = 0.24  R_J_ = 0.59  D_J_ = 0.18 | O_J_ = 0.11  R_J_ =0.74  D_J_ = 0.16 | O_J_ =0.36  R_J_ =0.09  D_J_ =0.55 | O_J_ = 0.1  R_J_ = 0.67  D_J_ = 0.24 | O_J_ = 0.27  R_J_ = 0.36  D_J_ = 0.36 | O_J_ = 0.14  R_J_ = 0.55  D_J_ = 0.32 |  |
| **Seamount X** | O_J_ = 0.13  R_J_ = 0.78  D_J_ = 0.09 | O_J_ = 0.13  R_J_ = 0.78  D_J_ = 0.09 | O_J_ =0.3  R_J_ =0.44  D_J_ =0.26 | O_J_ = 0.08  R_J_ = 0.92  D_J_ = 0 | O_J_ = 0.27  R_J_ = 0.13  D_J_ = 0.6 | O_J_ = 0.15  R_J_ = 0.77  D_J_ = 0.08 | O_J_ = 0.28  R_J_ = 0.44  D_J_ = 0.28 |

**S9 Table. The *pairwise pattern component* (PPC) values sensu Schmera et al. (2020) for the Juan de Fuca-Explorer Ridge complex vent sites based on the Jaccard-family of indices.** The PPCs include species *overlap* (O_J_), relativized species *replacement* (R_J_) and relativized species *richness difference* (D_J_). These values are illustrated in Fig 3C.

|  | **Explorer** | **Middle Valley** | **Endeavour** | **Co-Axial** | **Axial** | **N Cleft** |
| --- | --- | --- | --- | --- | --- | --- |
| **Middle Valley** | O_J_ = 0.47  R_J_ = 0.20  D_J_ = 0.33 |  |  |  |  |  |
| **Endeavour** | O_J_ = 0.51  R_J_ = 0.16  D_J_ = 0.33 | O_J_ = 0.49  R_J_ = 0.49  D_J_ = 0.02 |  |  |  |  |
| **Co-Axial** | O_J_ = 0.6  R_J_ = 0.34  D_J_ = 0.06 | O_J_ = 0.43  R_J_ = 0.20  D_J_ = 0.37 | O_J_ = 0.5  R_J_ = 0.13  D_J_ = 0.38 |  |  |  |
| **Axial** | O_J_ = 0.53  R_J_ = 0.22  D_J_ = 0.24 | O_J_ = 0.54  R_J_ = 0.36  D_J_ = 0.11 | O_J_ = 0.52  R_J_ = 0.39  D_J_ = 0.09 | O_J_ = 0.6  R_J_ = 0.1  D_J_ = 0.31 |  |  |
| **N Cleft** | O_J_ = 0.58  R_J_ = 0.39  D_J_ = 0.03 | O_J_ = 0.45  R_J_ = 0.2  D_J_ = 0.35 | O_J_ = 0.52  R_J_ = 0.13  D_J_ = 0.35 | O_J_ = 0.77  R_J_ = 0.19  D_J_ = 0.03 | O_J_ = 0.62  R_J_ = 0.1  D_J_ = 0.29 |  |
| **S Cleft** | O_J_ = 0.41  R_J_ = 0.19  D_J_ = 0.41 | O_J_ = 0.29  R_J_ = 0.08  D_J_ = 0.63 | O_J_ = 0.33  R_J_ = 0.04  D_J_ = 0.63 | O_J_ = 0.43  R_J_ = 0.2  D_J_ = 0.37 | O_J_ = 0.4  R_J_ = 0  D_J_ = 0.6 | O_J_ = 0.52  R_J_ = 0.07  D_J_ = 0.41 |

**S10 Table. The between-system *pairwise pattern component* (PPC) values sensu Schmera et al. (2020) for the vent sites in the Mariana region based on the Jaccard-family of indices.** The PPCs include species *overlap* (O_J_), relativized species *replacement* (R_J_) and relativized species *richness difference* (D_J_). Site pairs with no species *overlap* (O_J_ = 0) include the β-diversity partitions I_J_ (the intersection of nestedness and β-diversity) and RC_J_ (the relative compliment of nestedness in β-diversity), rather than D_J_ and R_J_, respectively. These values are illustrated in Fig 3D.

|  | **Alice Springs - Illium** | **Burke** | **Hafa Adai** | **Perseverance** | **Forecast** | **Snail** | **Archaean** | **Urashima-Pika** |
| --- | --- | --- | --- | --- | --- | --- | --- | --- |
| **Nikko** | O_J_ = 0.05  R_J_ = 0.51  D_J_ = 0.44 | O_J_ = 0.03  R_J_ = 0.65  D_J_ = 0.32 | O_J_ = 0.06  R_J_ = 0.57  D_J_ = 0.37 | O_J_ = 0  RC_J_ = 1  I_J_ = 0 | O_J_ = 0.07  R_J_ = 0.67  D_J_ = 0.27 | O_J_ = 0  RC_J_ = 1  I_J_ = 0 | O_J_ = 0.04  R_J_ = 0.92  D_J_ = 0.04 | O_J_ = 0  RC_J_ = 1  I_J_ = 0 |
| **Kasuga-2** | O_J_ = 0.05  R_J_ = 0.51  D_J_ = 0.44 | O_J_ = 0.06  R_J_ = 0.61  D_J_ = 0.33 | O_J_ = 0.06  R_J_ = 0.57  D_J_ = 0.37 | O_J_ = 0.04  R_J_ = 0.92  D_J_ = 0.04 | O_J_ = 0.07  R_J_ = 0.67  D_J_ = 0.27 | O_J_ = 0.03  R_J_ = 0.69  D_J_ = 0.28 | O_J_ = 0.09  R_J_ = 0.87  D_J_ = 0.04 | O_J_ = 0.04  R_J_ = 0.85  D_J_ = 0.12 |
| **NW Eifuku** | O_J_ = 0.14  R_J_ = 0.68  D_J_ = 0.18 | O_J_ = 0.1  R_J_ = 0.85  D_J_ = 0.05 | O_J_ = 0.15  R_J_ = 0.75  D_J_ = 0.1 | O_J_ = 0.1  R_J_ = 0.65  D_J_ = 0.26 | O_J_ = 0.14  R_J_ = 0.83  D_J_ = 0.03 | O_J_ = 0.11  R_J_ = 0.89  D_J_ = 0 | O_J_ = 0.1  R_J_ = 0.65  D_J_ = 0.26 | O_J_ = 0.06  R_J_ = 0.76  D_J_ = 0.18 |
| **Daikoku** | O_J_ = 0  RC_J_ = 1  I_J_ = 0 | O_J_ = 0  RC_J_ = 1  I_J_ = 0 | O_J_ = 0  RC_J_ = 1  I_J_ = 0 | O_J_ = 0  RC_J_ = 1  I_J_ = 0 | O_J_ = 0  RC_J_ = 1  I_J_ = 0 | O_J_ = 0  RC_J_ = 1  I_J_ = 0 | O_J_ = 0  RC_J_ = 1  I_J_ = 0 | O_J_ = 0  RC_J_ = 1  I_J_ = 0 |
| **Chamorro** | O_J_ = 0.03  R_J_ = 0.24  D_J_ = 0.73 | O_J_ = 0  RC_J_ = 1  I_J_ = 0 | O_J_ = 0.03  R_J_ = 0.28  D_J_ = 0.69 | O_J_ = 0  RC_J_ = 1  I_J_ = 0 | O_J_ = 0.04  R_J_ = 0.33  D_J_ = 0.63 | O_J_ = 0  RC_J_ = 1  I_J_ = 0 | O_J_ = 0  RC_J_ = 1  I_J_ = 0 | O_J_ = 0  RC_J_ = 1  I_J_ = 0 |
| **E Diamante** | O_J_ = 0.05  R_J_ = 0.65  D_J_ = 0.3 | O_J_ = 0.03  R_J_ = 0.79  D_J_ = 0.18 | O_J_ = 0.05  R_J_ = 0.72  D_J_ = 0.23 | O_J_ = 0.04  R_J_ = 0.86  D_J_ =0.11 | O_J_ = 0.06  R_J_ = 0.82  D_J_ = 0.12 | O_J_ = 0.03  R_J_ = 0.83  D_J_ = 0.14 | O_J_ = 0.04  R_J_ = 0.86  D_J_ = 0.11 | O_J_ = 0.03  R_J_ = 0.93  D_J_ = 0.03 |
| **NW Rota** | O_J_ = 0.09  R_J_ = 0.34  D_J_ = 0.57 | O_J_ = 0.03  R_J_ = 0.52  D_J_ = 0.45 | O_J_ = 0.1  R_J_ = 0.39  D_J_ = 0.52 | O_J_ = 0.1  R_J_ = 0.7  D_J_ = 0.2 | O_J_ = 0.12  R_J_ = 0.46  D_J_ = 0.42 | O_J_ = 0.07  R_J_ = 0.5  D_J_ = 0.43 | O_J_ = 0.05  R_J_ = 0.76  D_J_ = 0.19 | O_J_ = 0.04  R_J_ = 0.7  D_J_ = 0.26 |
| **Seamount X** | O_J_ = 0.08  R_J_ = 0.55  D_J_ = 0.38 | O_J_ = 0.06  R_J_ = 0.69  D_J_ = 0.26 | O_J_ = 0.08  R_J_ = 0.61  D_J_ = 0.31 | O_J_ = 0.08  R_J_ = 0.88  D_J_ =0.04 | O_J_ = 0.1  R_J_ = 0.71  D_J_ = 0.19 | O_J_ = 0.06  R_J_ = 0.73  D_J_ = 0.21 | O_J_ = 0.08  R_J_ = 0.88  D_J_ = 0.04 | O_J_ = 0.04  R_J_ = 0.93  D_J_ = 0.04 |

**S11 Table. The pairwise β-diversity values calculated using the Jaccard index (β_J_) and the Raup-Crick index (β_RC_ - in brackets) for the Mariana back-arc vent sites.** Positive and negative β_RC_ values indicate sites that are more and less different than random, respectively. Significant deviance from the null hypothesis of random assembly indicated with ‘**’. Values 0.9 and -0.9 represent the upper and lower bounds of the 95% confidence intervals. The β_RC_ values are used to generate the data points in Fig 4A.

|  | **Alice Springs -Illium** | **Burke** | **Hafa Adai** | **Perseverance** | **Forecast** | **Snail** | **Archaean** |
| --- | --- | --- | --- | --- | --- | --- | --- |
| **Burke** | 0.38  (-0.21) |  |  |  |  |  |  |
| **Hafa Adai** | 0.2  (-0.98**) | 0.22  (-0.99**) |  |  |  |  |  |
| **Perseverance** | 0.6  (-0.25) | 0.56  (-0.65) | 0.59  (-0.34) |  |  |  |  |
| **Forecast** | 0.47  (0.2) | 0.52  (0.22) | 0.45  (-0.33) | 0.63  (-0.17) |  |  |  |
| **Snail** | 0.44  (0.07) | 0.48  (0.01) | 0.47  (0.07) | 0.58  (-0.51) | 0.42  (-0.75) |  |  |
| **Archaean** | 0.65  (0.44) | 0.56  (-0.64) | 0.59  (-0.34) | 0.56  (-0.85) | 0.5  (-0.9**) | 0.58  (-0.49) |  |
| **Urashima-Pika** | 0.63  (0.71) | 0.64  (0.56) | 0.57  (-0.07) | 0.53  (-0.87) | 0.54  (-0.56) | 0.5  (-0.76) | 0.53  (-0.88) |

**S12 Table. The pairwise β-diversity values calculated using the Jaccard index (β_J_) and the Raup-Crick index (β_RC_ - in brackets) for the Mariana volcanic arc vent sites.** Positive and negative β_RC_ values indicate sites that are more and less different than random, respectively. Significant deviance from the null hypothesis of random assembly indicated with ‘**’. Values 0.9 and -0.9 represent the upper and lower bounds of the 95% confidence intervals. The β_RC_ values are used to generate the data points in Fig 4B.

|  | **Nikko** | **Kasuga-2** | **NW Eifuku** | **Daikoku** | **Chamorro** | **E Diamante** | **NW Rota** |
| --- | --- | --- | --- | --- | --- | --- | --- |
| **Kasuga-2** | 0.5  (-0.98**) |  |  |  |  |  |  |
| **NW Eifuku** | 0.82  (0.91**) | 0.86  (0.98**) |  |  |  |  |  |
| **Daikoku** | 0.56  (-0.94**) | 0.38  (-0.99**) | 0.87  (0.99**) |  |  |  |  |
| **Chamorro** | 0.79  (-0.52) | 0.94  (0.82) | 0.82  (-0.38) | 0.94  (0.89) |  |  |  |
| **E Diamante** | 0.83  (0.83) | 0.78  (0.5) | 0.77  (0.9**) | 0.75  (0.42) | 0.83  (-0.11) |  |  |
| **NW Rota** | 0.76  (-0.2) | 0.89  (0.83) | 0.64  (-0.92**) | 0.9  (0.92**) | 0.73  (-0.77) | 0.86  (0.79) |  |
| **Seamount X** | 0.87  (0.91**) | 0.87  (0.91**) | 0.7  (0.28) | 0.92  (0.99**) | 0.73  (-0.83) | 0.85  (0.95**) | 0.72  (-0.48) |

**S13 Table. The pairwise β-diversity values calculated using the Jaccard index (β_J_) and the Raup-Crick index (β_RC_ - in brackets) for the Juan de Fuca – Explorer Ridge complex vent sites.** Positive and negative β_RC_ values indicate sites that are more and less different than random, respectively. Significant deviance from the null hypothesis of random assembly indicated with ‘**’. Values 0.9 and -0.9 represent the upper and lower bounds of the 95% confidence intervals. The β_J_ values are illustrated in Fig 2. The β_RC_ values are used to generate the data points in Fig 4C.

|  | **Explorer** | **Middle Valley** | **Endeavour** | **Co-Axial** | **Axial** | **N Cleft** |
| --- | --- | --- | --- | --- | --- | --- |
| **Middle Valley** | 0.53  (-0.28) |  |  |  |  |  |
| **Endeavour** | 0.49  (-0.73) | 0.51  (0.97**) |  |  |  |  |
| **Co-Axial** | 0.4  (-0.99**) | 0.57  (-0.05) | 0.5  (-0.84) |  |  |  |
| **Axial** | 0.47  (-0.88) | 0.46  (0.43) | 0.48  (0.57) | 0.4  (-0.99**) |  |  |
| **N Cleft** | 0.42  (-0.99**) | 0.55  (-0.15) | 0.48  (-0.88) | 0.23  (-0.99**) | 0.38  (-0.99**) |  |
| **S Cleft** | 0.59  (-0.96**) | 0.71  (-0.28) | 0.67  (-0.78) | 0.57  (-0.99**) | 0.6  (-0.99**) | 0.48  (-0.99**) |

**S14 Table. The pairwise β-diversity values calculated using the Jaccard index (β_J_) and the Raup-Crick index (β_RC_ - in brackets) for the Mariana region – both the volcanic arc and back-arc.** Positive and negative β_RC_ values indicate sites that are more and less different than random, respectively. Significant deviance from the null hypothesis of random assembly indicated with ‘**’. Values 0.9 and -0.9 represent the upper and lower bounds of the 95% confidence intervals. The β_J_ values are illustrated in Fig 2. The β_RC_ values are used to generate the data points in Fig 4D.

|  | **AI** | **Bk** | **HA** | **Pv** | **Fn** | **Sn** | **Ar** | **UP** | **Nk** | **K2** | **NWE** | **Dk** | **Ch** | **ED** | **NWR** |
| --- | --- | --- | --- | --- | --- | --- | --- | --- | --- | --- | --- | --- | --- | --- | --- |
| **Bk** | 0.38  (-0.99**) |  |  |  |  |  |  |  |  |  |  |  |  |  |  |
| **HA** | 0.2  (-0.99**) | 0.22  (-0.99**) |  |  |  |  |  |  |  |  |  |  |  |  |  |
| **Pv** | 0.6  (-0.99**) | 0.56  (-0.99**) | 0.59  (-0.99**) |  |  |  |  |  |  |  |  |  |  |  |  |
| **Fn** | 0.47  (-0.99**) | 0.52  (-0.99**) | 0.45  (-0.99**) | 0.63  (-0.98**) |  |  |  |  |  |  |  |  |  |  |  |
| **Sn** | 0.44  (-0.99**) | 0.48  (-0.99**) | 0.47  (-0.99**) | 0.58  (-0.99**) | 0.42  (-0.99**) |  |  |  |  |  |  |  |  |  |  |
| **Ar** | 0.65  (-0.98**) | 0.56  (-0.99**) | 0.59  (-0.99**) | 0.56  (-0.99**) | 0.5  (-0.99**) | 0.58  (-0.99**) |  |  |  |  |  |  |  |  |  |
| **UP** | 0.63  (-0.97**) | 0.64  (-0.94**) | 0.57  (-0.99**) | 0.53  (-0.99**) | 0.54  (-0.99**) | 0.5  (-0.99**) | 0.53  (-0.99**) |  |  |  |  |  |  |  |  |
| **Nk** | 0.95  (0.99**) | 0.97  (0.99**) | 0.94  (0.99**) | 1  (1**) | 0.93  (0.97**) | 1  (1**) | 0.96  (0.96**) | 1  (1**) |  |  |  |  |  |  |  |
| **K2** | 0.95  (0.99**) | 0.94  (0.98**) | 0.94  (0.99**) | 0.96  (0.97**) | 0.93  (0.97**) | 0.97  (0.99**) | 0.91  (0.75) | 0.96  (0.98**) | 0.5  (-0.99**) |  |  |  |  |  |  |
| **NWE** | 0.86  (0.99**) | 0.9  (0.99**) | 0.85  (0.97**) | 0.9  (0.92**) | 0.86  (0.93**) | 0.89  (0.99**) | 0.9  (0.91**) | 0.94  (0.99**) | 0.82  (0.13) | 0.86  (0.6) |  |  |  |  |  |
| **Dk** | 1  (1**) | 1  (1**) | 1  (1**) | 1  (1**) | 1  (1**) | 1  (1**) | 1  (1**) | 1  (1**) | 0.56  (-0.99**) | 0.38  (-0.99**) | 0.87  (0.8) |  |  |  |  |
| **Ch** | 0.97  (0.97**) | 1  (1**) | 0.97  (0.93**) | 1  (1**) | 0.96  (0.85) | 1  (1**) | 1  (1**) | 1  (1**) | 0.79  (-0.82) | 0.94  (0.53) | 0.82  (-0.84) | 0.94  (0.65) |  |  |  |
| **ED** | 0.95  (1**) | 0.97  (1**) | 0.95  (0.99**) | 0.96  (0.99**) | 0.94  (0.99**) | 0.97  (1**) | 0.96  (0.99**) | 0.97  (0.99**) | 0.83  (0.11) | 0.78  (-0.43) | 0.77  (-0.13) | 0.75  (-0.57) | 0.83  (-0.64) |  |  |
| **NWR** | 0.91  (0.9**) | 0.97  (0.99**) | 0.90  (0.79) | 0.9  (0.47) | 0.88  (0.49) | 0.93  (0.89) | 0.95  (0.89) | 0.96  (0.94**) | 0.76  (-0.75) | 0.89  (0.39) | 0.64  (-0.99**) | 0.9  (0.57) | 0.73  (-0.92**) | 0.86  (0.18) |  |
| **SX** | 0.93  (0.99**) | 0.94  (0.99**) | 0.92  (0.99**) | 0.92  (0.86) | 0.9  (0.93**) | 0.94  (0.99**) | 0.92  (0.86) | 0.96  (0.99**) | 0.87  (0.4) | 0.87  (0.41) | 0.7  (-0.78) | 0.92  (0.91**) | 0.73  (-0.96**) | 0.85  (0.38) | 0.72  (-0.89) |

1. Kojima S. Deep-sea chemoautosynthesis-based communities in the northwestern Pacific. J Oceanogr. 2002;58:343-63.

2. de Ronde C, Chadwick W, Ditchburn R, Embley R, Tunnicliffe V, Baker E, et al. Submarine sulfur lakes: degassing of intraoceanic arc volcanoes. In: D Rouwet RT, J Vandelbroulemuck and B Christenson, editor. Volcanic Lakes. Berlin: Springer 2015. p. 261 - 88.

3. Fujikura K, S. Tsuchida HU, J. Ishibashi, W. Gaze, Y. Maki. Investigation of the Deep-sea chemosynthetic ecosystem and submarine Volcano at the Kasuga 2 and 3 Seamounts in the Northern Mariana Trough, Western Pacific. JAMSTEC J Deep Sea Res. 1998;14:127 - 37.

4. Limén H, Juniper SK, Tunnicliffe V, Clément M. Benthic community structure on two peaks of an erupting seamount: Northwest Rota-1 Volcano, Mariana Arc, western Pacific. Cah Biol Mar. 2006;47:457-64.

5. Tunnicliffe V, Tyler J, Dower JF. Population ecology of the tonguefish *Symphurus thermophilus* (Pisces; Pleuronectiformes; Cynoglossidae) at sulphur-rich hydrothermal vents on volcanoes of the northern Mariana Arc. Deep Sea Res 2 Top Stud Oceanogr. 2013;92(0):172-82.

6. Stevens CJ, Juniper SK, Limén H, Pond DW, Metaxas A, Gélinas Y. Obligate hydrothermal vent fauna at East Diamante submarine volcano (Mariana Arc) exploit photosynthetic and chemosynthetic carbon sources. Mar Ecol Prog Ser. 2015;525:25-39.

7. Tunnicliffe V, Botros M, De Burgh ME, Dinet A, Johnson HP, Juniper SK, et al. Hydrothermal vents of Explorer Rdge, northeast Pacific. Deep Sea Res A. 1986;33(3):401-12.

8. Grehan AJ, Juniper SK. Clam distribution and subsurface hydrothermal processes at Chowder Hill (Middle Valley), Juan de Fuca Ridge. Mar Ecol Prog Ser. 1996;130:105-15.

9. Juniper SK, Tunnicliffe V, Southward EC. Hydrothermal vents in turbidite sediments on a Northeast Pacific spreading centre: organisms and substratum at an ocean drilling site. Can J Zool. 1992;70(9):1792-809.

10. Levin LA, Mendoza GF, Konotchick T, Lee R. Macrobenthos community structure and trophic relationships within active and inactive Pacific hydrothermal sediments. Deep Sea Res 2 Top Stud Oceanogr. 2009;56(19–20):1632-48.

11. Tunnicliffe V, Cordes EE. The Tubeworm Forests of Hydrothermal Vents and Cold Seeps. In: Rossi S, Bramanti L, editors. Perspectives on the Marine Animal Forests of the World. Cham: Springer International Publishing; 2020. p. 147-92.

12. Sarrazin J, Juniper SK. Biological characteristics of a hydrothermal edifice mosaic community. Mar Ecol Prog Ser. 1999;185:1-19.

13. Lelièvre Y, Sarrazin J, Marticorena J, Schaal G, Day T, Legendre P, et al. Biodiversity and trophic ecology of hydrothermal vent fauna associated with tubeworm assemblages on the Juan de Fuca Ridge. Biogeosciences. 2018;15(9):2629-47.

14. Tunnicliffe V, Embley RW, Holden JF, Butterfield DA, Massoth GJ, Juniper SK. Biological colonization of new hydrothermal vents following an eruption on Juan de Fuca Ridge. Deep Sea Res 1 Oceanogr Res Pap. 1997;44(9–10):1627-44.

15. Marcus J, Tunnicliffe V, Butterfield DA. Post-eruption succession of macrofaunal communities at diffuse flow hydrothermal vents on Axial Volcano, Juan de Fuca Ridge, Northeast Pacific. Deep Sea Res 2 Top Stud Oceanogr. 2009;56(19–20):1586-98.

16. Limén H, Levesque C, Kim Juniper S. POM in macro-/meiofaunal food webs associated with three flow regimes at deep-sea hydrothermal vents on Axial Volcano, Juan de Fuca Ridge. Mar Biol. 2007;153(2):129-39.

17. Milligan B. Geological effects on faunal distributions on the Cleft Segment, Juan de Fuca Ridge. Masters Thesis, Univ Victoria, Canada. 1993; 129pp.

18. Sarrazin J, Juniper SK, Massoth G, Legendre P. Physical and chemical factors influencing species distributions on hydrothermal sulfide edifices of the Juan de Fuca Ridge, northeast Pacific. Mar Ecol Prog Ser. 1999;190:89-112.
